# Aflatoxins in Cereals and Cereal-Based Products: Occurrence, Toxicity, Impact on Human Health, and Their Detoxification and Management Strategies

**DOI:** 10.3390/toxins14100687

**Published:** 2022-10-06

**Authors:** Pradeep Kumar, Akansha Gupta, Dipendra Kumar Mahato, Shikha Pandhi, Arun Kumar Pandey, Raveena Kargwal, Sadhna Mishra, Rajat Suhag, Nitya Sharma, Vivek Saurabh, Veena Paul, Manoj Kumar, Raman Selvakumar, Shirani Gamlath, Madhu Kamle, Hesham Ali El Enshasy, Jawahir A. Mokhtar, Steve Harakeh

**Affiliations:** 1 Department of Botany, University of Lucknow, Lucknow 226007, India; 2 Applied Microbiology Laboratory, Department of Forestry, North Eastern Regional Institute of Science and Technology, Nirjuli 791109, India; 3 Department of Dairy Science and Food Technology, Institute of Agricultural Sciences, Banaras Hindu University, Varanasi 221005, India; 4 CASS Food Research Centre, School of Exercise and Nutrition Sciences, Deakin University, Burwood, VIC 3125, Australia; 5 MMICT&BM(HM), Maharishi Markandeshwar (Deemed to be University), Mullana, Ambala 133207, India; 6 Department of Processing and Food Engineering, College of Agricultural Engineering and Technology, Chaudhary Charan Singh Haryana Agricultural University, Hisar 125004, India; 7 Faculty of Agricultural Sciences, GLA University, Mathura 281406, India; 8 Faculty of Science and Technology, Free University of Bozen-Bolzano, Piazza Università 5, 39100 Bolzano, Italy; 9 Food and Bioprocess Engineering Laboratory, Centre for Rural Development and Technology, Indian Institute of Technology Delhi, New Delhi 110016, India; 10 Division of Food Science and Postharvest Technology, ICAR—Indian Agricultural Research Institute, New Delhi 110012, India; 11 Chemical and Biochemical Processing Division, ICAR—Central Institute for Research on Cotton Technology, Mumbai 400019, India; 12 Centre for Protected Cultivation Technology, ICAR-Indian Agricultural Research Institute, Pusa Campus, New Delhi 110012, India; 13 Institute of Bioproduct Development, Universiti Teknologi Malaysia (UTM), Skudai 81310, Malaysia; 14 City of Scientific Research and Technology Applications, New Burg Al Arab, Alexandria 21934, Egypt; 15 Department of Medical Microbiology and Parasitology, Faculty of Medicine, King Abdulaziz University Hospital, Jeddah 21589, Saudi Arabia; 16 Vaccines and Immunotherapy Unit, King Fahad Medical Research Center, King Abdulaziz University, Jeddah 21589, Saudi Arabia; 17 King Fahd Medical Research Center, King Abdulaziz University, Jeddah 21589, Saudi Arabia; 18 Yousef Abdul Latif Jameel Scientific Chair of Prophetic Medicine Application, Faculty of Medicine (FM), King Abdulaziz University, Jeddah 21589, Saudi Arabia

**Keywords:** aflatoxins, food contamination, health issues, detection technique, conventional and novel management strategies

## Abstract

Cereals and cereal-based products are primary sources of nutrition across the world. However, contamination of these foods with aflatoxins (AFs), secondary metabolites produced by several fungal species, has raised serious concerns. AF generation in innate substrates is influenced by several parameters, including the substrate type, fungus species, moisture content, minerals, humidity, temperature, and physical injury to the kernels. Consumption of AF-contaminated cereals and cereal-based products can lead to both acute and chronic health issues related to physical and mental maturity, reproduction, and the nervous system. Therefore, the precise detection methods, detoxification, and management strategies of AFs in cereal and cereal-based products are crucial for food safety as well as consumer health. Hence, this review provides a brief overview of the occurrence, chemical characteristics, biosynthetic processes, health hazards, and detection techniques of AFs, along with a focus on detoxification and management strategies that could be implemented for food safety and security.

## 1. Introduction

Crops, mainly cereal grains, serve as a major source of energy and nutrition in the human diet worldwide. Cereals are generally consumed as raw or cooked grains or in the form of processed products such as flour, semolina, bread, and cookies. Often, cereal crops and their byproducts are also used as animal feed for livestock and poultry, which are eventually rendered as sources of dairy, poultry, and meat products for human consumption. According to the Food and Agriculture Organization (FAO), one-fourth of the world’s total cereal crop production is contaminated with mycotoxins [1,2]. In the last few decades, the number of cases of mycotoxicosis in humans has increased due to the consumption of food contaminated with one or more mycotoxins, which has ultimately also affected the sustainability of agribusinesses [3,4,5]. The acute toxicity and carcinogenic effects of mycotoxins and their contamination in cereals and cereal-based products pose a serious food safety and security concern for humans, along with huge economic losses [6].

**Figure 1:**
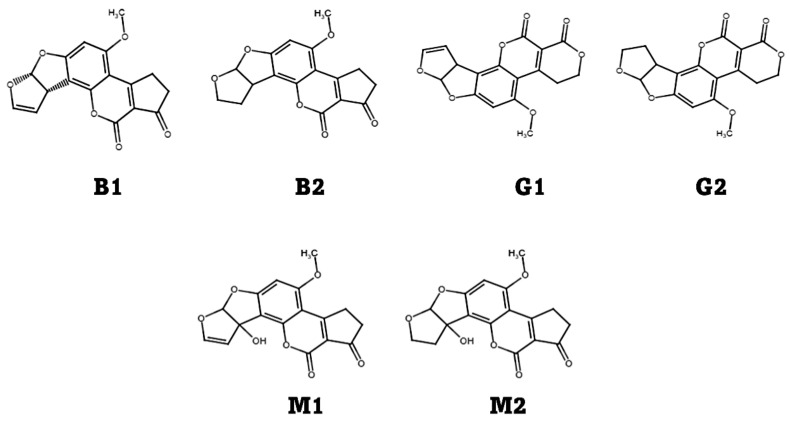
Chemical structures of different types of aflatoxins.

Mycotoxins, such as fumonisins, ochratoxin A, trichothecenes (type A and B), aflatoxins, and patulin, are secondary metabolites secreted by several fungal species, mainly *Aspergillus*, *Fusarium*, *Penicillium*, and *Alternaria*, which often contaminate cereal crops at the farm level during handling, transportation, and/or storage [6,7,8,9,10,11,12,13,14]. Most mycotoxins are resistant to food processing techniques due to their thermostable nature; hence, their contamination in processed products must be checked before consumption. According to Khaneghah et al. [15], total aflatoxin (AF) is the most prevalent mycotoxin contamination worldwide after ochratoxin, zearalenone, and deoxynivalenol, found in cereals and cereal products such as whole grains, bread, cornflakes, breakfast cereals, and pasta products. As secondary metabolites of fungal strains (*Aspergillus parasiticus*, *Aspergillus flavus*, and *Aspergillus nomius*), AFs (B1, B2, G1, and G2) develop on a variety of food and feed materials during growing, harvesting, storing, and shipping processes [16,17]. Out of 20 known AFs, 4 (B1, B2, G1, and G2) have been identified as major contaminants in cereals, such as peanuts, maize, rice, barley, and sorghum, and in their products [18]. AFB1 has the highest prevalence in cereal products of all AFs [19]. Under UV light (365 nm), aflatoxins emit blue (B1 and B2) or green (G1) and green-blue (G2) fluorescence [20]. These different groups of aflatoxins differ in their structures at the molecular level; for instance, the cyclopentane ring is identical in group B aflatoxins (B1 and B2) and group M aflatoxins (M1 and M2), while group G aflatoxins (G1 and G2) contain a lactone ring (Figure 1). Based on toxicity, aflatoxin types can be arranged as B1 > G1 > B2 > G2 [21]. Andrade and Caldas [22] reported that 37.6% of 18,097 tested cereal samples worldwide were contaminated with at least one AF, according to 89 publications.

The World Health Organization (WHO) has recognized AFs as a global food safety concern, and rural populations in developing countries are at an especially high risk of AF exposure [23]. The International Agency for Research on Cancer (IARC) classified AFs as group 1 carcinogens due to their toxic, carcinogenic, mutagenic, teratogenic, and immunotoxic nature [24,25]. Due to serious health complications in humans and animals, several countries have implemented strict regulations to prevent AF contamination in food and feed. According to the European Commission Regulation, the maximum permissible limit for total AFs and AFB1 in all cereals and their derived products intended for direct consumption should not be more than 4 µg/kg and 2 µg/kg, respectively [26,27]. However, 20 µg/kg is the maximum acceptable limit for AFs in the United States [28]. In some countries (such as in the EU), there are limits for raw cereals and processed products. Apart from limiting the maximum acceptable limit, several innovative techniques and management practices are also adopted at pre- and post-harvest processing levels to control and/or prevent aflatoxin contamination in cereals and their derived products. Though several reports and publications are available on control and management strategies for aflatoxin contamination in food and feed, a gap exists in the literature with a focus on novel and environmentally friendly approaches. Hence, the present review aims to provide an overview of AF contamination in cereals and cereal-based products and suggests the best environmentally friendly practices that could be implemented to ensure food safety as well as prevent possible AF outbreaks. 

## 2. Major Source and Occurrence of Aflatoxins

AFs are one of the major mycotoxins produced in cereals by several species of *Aspergillus*, mainly *A. flavus*, *A. nomuius*, *A. parasiticus*, and *A. astellatus*. Other AF-producing species, such as *A. bombycis*, *A. ochraceoroseus*, and *A. pseudotamariii*, have been identified using advanced genome sequencing techniques [29]. The warm and humid environment of tropical and subtropical regions is favorable for the growth of these fungal species [30]. Out of different AF types, B1, B2, G1, and G2 are found in plant-based foods, including cereal grains, while the metabolites of type AFB1, i.e., AF M1 and M2, are especially found in foods of animal origin [31]. *Aspergillus* species such as *A. flavus* and *A. pseudotamarii* are mainly responsible for the production of type B AFs only, as they cannot produce type G aflatoxins due to the absence of 0.8 to 1.5 kb in the 28-gene cluster responsible for AF biosynthesis. However, other *Aspergillus* species, such as *A. parasiticus*, *A. nomius*, and *A. bombycis*, are capable of producing all four major AFs. AFs M1 and M2 are the hydrated metabolites of AFs B1 and B2, respectively, and their contamination is usually observed in products derived from animals when exposed to feed contaminated with AF B1 and/or B2. The presence of AF M1 at a higher concentration has been reported in human breast milk from countries such as Australia and Thailand, which shows the risk of aflatoxicosis in infants [32].

Among cereals, AF contamination is frequently observed in crops such as rice and corn compared to other cereals [33]. AFB1 contamination in rice has been reported in several countries, including China, Egypt, India, Iran, Malaysia, Nepal, Pakistan, the Philippines, the United Kingdom, and the United States [34]. The improper drying of rice grains, when the moisture content is >14%, is mainly responsible for fungal growth, which later causes the discoloration of grains and/or husks, and the production of toxic secondary metabolites, such as AF, and ultimately leads to the complete deterioration of edible-grain quality [2]. Climate changes, including temperature, moisture content, water activity (a_w_), type of soil, and storage conditions, are major factors influencing fungal growth and their ability to produce AFs in cereals crops [35,36]. Lv et al. [37] reported that the maximum AFB1 production occurs at a temperature of 33 °C and water activity (a_w_) of 0.96, whereas Gizachew et al. [38] reported that temperatures ranging from 28–37 °C at 0.92–0.96 a_w_ led to the optimal growth of fungi (*A. flavus* and *A. parasiticus*) on polished rice. According to Battilani et al. [39], every 2 °C increase in temperature as a result of climate change could increase the emergence of AFB1 in various regions of Europe, such as Albania, Bulgaria, Cyprus, Greece, Italy Spain, Portugal, and Turkey. Furthermore, in the next 30 years, the risk of AF contamination in maize crops is expected to rise in Europe due to changing climatic conditions that are favorable for AF-producing fungi such as *A. flavus* [40]. Further, the type of AFs detected in various food sources around the world between 2010 and 2022 and the method of detection using various techniques are presented in Table 1. Aflatoxins have been found in a variety of cereals and their products, including barley-based products, corn, corn bran, corn flour, corn ingredients, corn-based opaque beers, multigrain-cereal baby foods, pearl millet, rice, rice-based baby foods, rice flour, sorghum, sorghum beer, sorghum malt, sorghum-based products, wheat and wheat-based baby foods, wheat bran, wheat flour, and wheat-based products. Various research studies on aflatoxins in cereals and their byproducts have been conducted, and they were detected in nearly every country, such as Africa, Bangladesh, Brazil, Burkina Faso and Mozambique, China, Colombia, Costa Rica, Egypt, Ethiopia, Ghana, India, Iran, Kenya, Mediterranean area, Namibia, Niger, Pakistan, Peru, Serbia, South Africa, South Korea, Spain, Tanzania, Tanzania, Thailand, Togo, Tunisia, Turkey, Uganda, Vietnam, and Zimbabwe.

## 3. Chemistry and Biosynthesis of Aflatoxins

All AFs are heterocyclic compounds with a basic benzene ring, with minor differences in the occurrence of double bonds and ketonic groups, as well as the presence of hydroxy groups in the metabolites, with hydroxylation sites varying from one molecule to another. These structures imply a low water solubility and an easy epoxidation reaction, which are expected to impact both elimination and lethality. The most common and potent human health concern in the world, AFB1, contains a unique double bond in the cyclic ring, which is also seen in G1 and M1. AFB1 must be epoxidized to AFB1 2,3-epoxide in order to be functional. The toxin is biotransformed into the less lethal AFs M1 and G1 by microsomal cytochrome P450 (CYP450) monooxygenases [92,93]. A double bond at carbons 8 and 9 in AFs B1 and G1 facilitates the synthesis of epoxide, a more lethal version of AFs B1 and G1, but not in AFs B2 and G2. The dihydroxy derivatives of B1 and G1 were identified as AFs B2 and G2, respectively. AF M1 is a 4-hydroxy AFB1, whereas AF M2 is a 4-dihydroxy AF B2. B1 and G1 are hydrogenated to produce B2 and G2, respectively [92,94].

**Figure 2:**
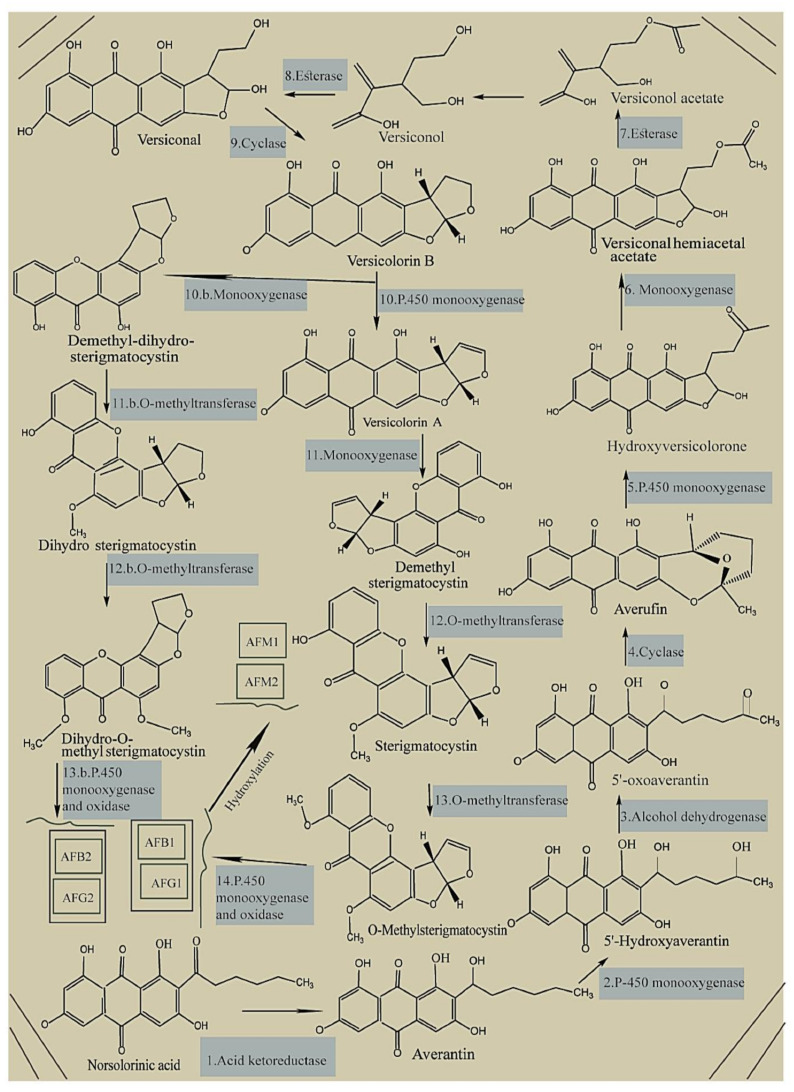
Biosynthesis pathway of aflatoxins. Reprinted with permission from Nazhand et al. [112].

The primary substrate of hexanoyl is transformed into a polyketide by a polyketide synthase and two fatty acid synthases during the biosynthesis of aflatoxins in crops by *Aspergillus flavus* and *Aspergillus parasiticus* [95,96], followed by the conversion of the polyketide to norsolorinic acid anthrone by polyketide synthase; thereafter, norsolorinic acid anthrone is converted to norsolorinic acid (NOR), which is the first stable forerunner of aflatoxins (Figure 2) [97,98]. Then, the reductase enzyme converts NOR to averantin [99], and 5′-hydroxyaverantin (HAVN) is created from averantin using the monooxygenase enzyme [100]. Further, dehydrogenase converts HAVN to 5′-oxoaverantin (OAVN), which forms averufin (AVF) using cyclase [101,102,103], followed by the Baeyer–Villiger reaction, forming hydroxyversicolorone (HVN) from AVF [104]. Next, HVN is oxidized to versiconal hemiacetal acetate (VHA), which is further converted to versiconol acetate (VOAc) and then to versiconol (VOH) [105]. Using esterase, VOH forms versiconal, which is then transformed into versicolorin B by cyclase [106]. Furthermore, versicolorin B is converted to versicolorin A and dimethyl-dihydro-sterigmatocystin (DMDHST). Next, versicolorin A forms dimethyl-sterigmatocystin (DMST), and DMDHST forms dihydro-sterigmatocystin (DHST) [107,108]. Thereafter, O-methyltransferases transform the intermediates of DMST and DHST to sterigmatocystin (ST) and dihydro-O-methylsterigmatocystin (DHOMST), respectively, playing a crucial role in the biosynthesis of AFs [109]. Next, ST produces O-methylsterigmatocystin (OMST), which, along with DHOMST, finally produces AFs [110,111].

**Figure 3:**
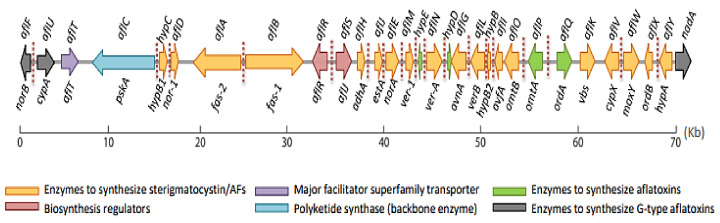
Aflatoxin gene cluster organization, including old and new cluster gene nomenclatures (red dotted lines represent the binding sites of AflR in the above pathway). Figure reprinted from Caceres et al. [96].

In Aspergilli, DNA information is structured into eight chromosomes, with genes relevant for AF production found in the 54^th^ cluster, 80 kb from chromosome 3’s telomere [113]. This cluster contains 30 genes, and aflR and aflS are the major regulators of its activation [114,115] (Figure 3). In *Aspergillus flavus* and *Aspergillus parasiticus*, the AF gene cluster has been extensively explored. The clustered genes of the two fungal species are 90–99% homologous [109]. The ability to create B- and G-type AFs is one of the key differences between the two species. *Aspergillus flavus* produces mostly B-type AFs (B1 and B2), while *Aspergillus parasiticus* produces both B- and G-type AFs (B1, B2, G1, and G2). The functional genes aflU, aflF, and nadA, which code for a cytochrome P-450 monooxygenase, an aryl alcohol dehydrogenase, and an oxidase, respectively, are involved in the formation of G-type AFs [116,117]. Experiments on the potential of *Aspergillus nidulans* to make sterigmatocystin have also aided in the understanding of the aflatoxin cluster. Indeed, there is 55–75% similarity between *Aspergillus parasiticus* and *Aspergillus nidulans* clusters [109].

## 4. Health Effects and Mechanism of Toxicity

Human exposure to AFs can occur at any stage of life, either directly by the ingestion of AF-contaminated food or indirectly due to the intake of foods (milk, egg, meat, etc.) derived from livestock previously exposed to AF-contaminated feed [118,119]. When ingested, AF passes through the metabolic process in mammals and remains unaltered, and it later accumulates in the tissues [21]. It is now well established that, apart from cancer, AFs also cause acute and severe chronic diseases. Initially, the carcinogenicity of AFs was identified and associated with the liver, which first metabolizes them and produces reactive intermediary metabolites. However, subsequent epidemiological and animal studies revealed their carcinogenic effects on other organs, including the kidney, pancreas, bladder, bone, viscera, and central nervous system [120]. Evidence has shown that AFB1-mediated oxidative stress is equally or even more responsible for AF-induced genotoxicity. The second-most documented toxicological effect of AFs is probably immunotoxicity, and its mechanisms of action (immunosuppressive and immunostimulatory actions) have been extensively illustrated [121]. Apart from the above, malnutrition, disease, impaired child growth, retardation of physical and mental maturity, reproduction, nervous system diseases, etc., are some other AF-induced acute and chronic health issues reported in mammals. However, further studies are required to demonstrate their precise mechanisms of action [122].

Different mechanisms of action are responsible for the various toxicological effects of AFs, but most of them are not fully understood yet. Since the AF discovery, AFB1 has been a major focus, as it is responsible for forming the intermediate metabolite AFB1-exo-8,9 epoxide (AFBO) [123]. This intermediate metabolite is a highly unstable molecule that reacts with different cellular macromolecules, including nucleic acids, proteins, and phospholipids, and thereby induces various disruptions at the genetic, metabolic, signaling, and cellular structure levels [124,125]. However, several studies have also evidenced that AFB1 has equivalent or even more effects on the integrity of cell function due to induced oxidative stress (OS) [122,126,127]. 

## 5. Effects of Environmental Factors on Aflatoxin Production

Environmental factors, such as water activity (a_w_), temperature, and pressure, are critical factors for *A. flavus* growth and AF accumulation. In addition, pH, CO_2_ levels, and light exposure have also been shown to significantly impact fungal growth and AF production [128,129,130]. Lowering a_w_ in foods inhibits microbiological proliferation and biochemical processes, extending the food product shelf life [131]. The proportions of AF-producing microbial communities that develop during the pre-harvest step have a significant impact on the post-harvest step, and the impact of prolonged harvesting on contamination is especially critical when rain damages crops before or during harvesting [132]. Variables for germination, proliferation, and AF production by *A. flavus* and *A. parasiticus* reveal that proliferation happens on a broader spectrum than production, with AF production occurring over an even smaller range than growth. The ideal conditions for AF production in these two microbial species (*A. flavus* and *A. parasiticus*) are 33 °C and 0.99 a_w_; on the contrary, the optimal conditions for production are 35 °C and 0.95 a_w_ [133]. Pitt and Miscamble [134] reported that the influence of environmental conditions on the development of *A. flavus*, *A. parasiticus*, and *A. oryzae* was comparable, exhibiting minima at 0.82 a_w_/25 °C and 0.81a_w_/30 and 37 °C. However, there was no assessment of AF production comparing *A. flavus* and *A. parasiticus* in the investigative study. Giorni et al. [135] reported that moist maize supplemented with 25% CO_2_ is adequate for the inhibition of *A. flavus* germination, while about 50% CO_2_ was necessary to substantially reduce AF formation. Managing hydrogen peroxide [136] and carbon dioxide [137] concentration through food processing and storage is, thus, an appropriate management method for avoiding *A. flavus* infestation and consequent AF production.

## 6. Detection Techniques

Various chromatographic, spectrometric, and sensor-based techniques have become prevalent for the sensitive detection and quantification of AFs in cereals and cereal-based products. Among chromatographic techniques, liquid chromatography (LC), thin-layer chromatography (TLC), and high-performance liquid chromatography (HPLC), along with numerous detectors, have been utilized for cereal-based food and feed products. HPLC is the most sensitive and accurate technique. Samples for HPLC are prepared through dry, wet, or cryogenic grinding, solvent extraction, solid-phase extraction, immunoaffinity column clean-up, and supercritical fluid extraction [138]. Namjoo, et al. [139] proposed the use of an HPLC column with a mobile phase of water–methanol–acetonitrile (60:30:15, *v*/*v*/*v*) coupled with a fluorescence detector to quantify the levels of AF B1_,_ B2_,_ G1_,_ and G2, in wheat silos in Golestan Province of Iran. AF presence was found in ten out of thirty-four wheat samples, all of which were below the permitted levels in Iran (15 µg/kg). The highest levels found in samples for total AFs, AFB1, AFB2, AFG1, and AFG2 were 7.08, 6.91, 0.29, 1.37, and 0.23 µg/kg, respectively. Likewise, AFB1 was analyzed in wheat and rice flour samples with immunoaffinity column clean-up using post-column photochemical derivatization and HPLC-FLD, where the limits of determination of AFB1 were observed to be 0.015 and 0.05 µg/kg, respectively. Spectrophotometric detection methods are preferred over these chromatographic methods, as they help to directly screen large lots of cereals to segregate putatively contaminated kernels. Near-infrared spectroscopy, fluorescent spectroscopy, and multispectral and hyperspectral imaging are some commonly used techniques that allow the inexpensive and rapid detection of AFs in cereal grains such as wheat, maize, corn, sorghum, rice, etc. [140,141,142]. For instance, Liu et al. [143] applied surface-enhanced Raman spectroscopy (SERS) combined with the QuEChERS sample pre-treatment technique for the trace-level detection of aflatoxin B1 in wheat and corn with high sensitivity and strong specificity. The method showed a good linear-log relationship between the Raman signal intensity of AFB1 in the 1–1000 µg/L concentration range with a limit of detection of 0.85 µg/kg. SERS is a rapid and sensitive technique for detecting trace mycotoxins. However, the sample preparation of SERS substrates for the quantitative analysis of foods is relatively challenging. Collectively, chromatographic–spectrometric techniques offer the advantage of a “dilute and shoot” approach, where sample extracts are analyzed without clean-up, and with the added advantage of multi-mycotoxin analysis, whereby a range of mycotoxins can be analyzed in the same sample analysis [144].

Lately, nanomaterial-based sensors with versatile properties have been observed to be highly selective and specific in terms of the trace detection of AFs in food samples. These also cater to the limitations posed by other conventionally used detection methods, as mentioned previously. Many nanomaterials have been used for the immobilization of biomolecules as signal generators or fluorescent quenchers or for signal amplification in AFB1 detection [145,146]. Recently, graphene oxide and gold nanowires were used as novel nanomaterials to develop fluorescence resonance energy transfer (FRET) and electrochemical-based sensors for the detection of AFs [147]. Further, electrochemical aptasensors based on carbon-based nanocomposites have also demonstrated an exceptional ability to detect and quantify AF concentrations in cereal-based foods [148,149,150].

## 7. Detoxification and Management Strategies

### 7.1. Conventional Agricultural Practices

AF production is stimulated by drought, stress, and high temperatures due to improper cultivation practices and post-harvest management [31]. The execution of modern agricultural practices, such as good agricultural practices (GAPs), good manufacturing practices (GMPs), good storage practices (GSPs), and preharvest management strategies [2,151], might be helpful for the prevention of AF contamination in grains. Various breeding programs have experimented on maize for the development of AF-resistant lines using inbred lines resistant to AF-resistant germplasm and the associated resistance proteins for host resistance, proteomic and molecular breeding, and genetic engineering, employing potential biochemical markers and genes for resistance [152,153,154,155,156]. These approaches could help manage AFs in pre- and post-harvest conditions. The AF content and the chances of contamination under field conditions also depend on the sowing time and cultivation region. Higher contamination was observed in late-cultivated compared to early-cultivated maize crops [157,158]. Further, several physical, chemical, and biological methods are being applied for the decontamination of AFs in cereal and cereal-based products. 

### 7.2. Physical Methods

Physical methods can be applied through washing, cleaning, density segregation, sieving, dehulling, adsorption, thermal treatments, and the use of gamma, UV, and visible light radiation [159]. Generally, agricultural produce goes through a common practice of sorting. This practice is supported by the fact that mycotoxin contamination tends to have a skewed distribution, with the majority of toxins found in a small number of grains or kernels [160]. Matumba et al. [161] performed experiments to evaluate the effectiveness of various physical methods in mycotoxin-contaminated white maize. Out of the experimented methods, hand sorting was found to be the most effective in reducing AFs, which removed about 95% of the AF content in white maize. However, sorting practices have been improved, and better technologies are being used for better accuracy and speed. Pearson et al. [162] used a high-speed dual-wavelength method for sorting yellow corn using absorbances at 750 and 1200 nm. It correctly identified >99% of AF-contaminated kernels, which reduced about 81% of AFs. 

Irradiation is a method in which a product is penetrated with ionizing radiation [163]. Silva et al. [164] investigated gamma irradiation (^60^CO) as a rice storage option. They found that it effectively controlled *Penicillium* spp. and *Aspergillus* spp., resulting in a reduction in AF levels in stored grains. The application of low-temperature radiofrequency plasma was used to degrade AFs, and the quantities of AFB1 (2–50 g/L) in the solution were reduced by 88% [165]. In addition, aflatoxigenic molds were successfully managed at different stages of germination, sporulation, and growth by 5 kGy gamma irradiation. The inhibition efficacy was observed to be dose-dependent in the feed sample. A 10 kGy dose reduced about 90% of AFB1, while a 5 kGy dose reduced 70% of AFB1 in artificially contaminated maize samples [166]. Besides this, ultraviolet (UV) irradiation for 1–3 h followed by 6 months of storage revealed 22–79% fewer fungal colonies as well as lower AF content in brown, black, and red rice grains [167]. Furthermore, pulse light (PL) is a non-thermal approach used for the inhibition of AFs. *A. flavus*-contaminated rough rice and rice bran were treated with PL at 0.52 J/cm^2^/pulse for varying time durations. The treatment removed 75.0% of AFB1 and 39.2% of AFB2 in rough rice and 90.3% of AFB1 and 86.7% of AFB2 in rice bran when treated for 80 s and 15 s, respectively [168].

Besides this, extrusion and baking are two of the most utilized heat-based procedures in the food sector for minimizing mycotoxins. Extrusion is a high-temperature, short-time procedure involving heat, humidity, and mechanical force. It alters raw materials in such a way that they take on new shapes and structures with distinct functional and nutritional properties. In the food industry, this heat treatment is used to produce biscuits, pasta, ready-to-eat cereals, snacks, pellets, etc. The presence or absence of additives, moisture content, and temperature all appear to alter the effect of extrusion on AF levels. Extrusion alone was demonstrated to reduce AF levels by 50–80%, and adding ammonia, either as hydroxide (0.7–1.0%) or as bicarbonate (0.4%), increased the AF reduction to more than 95% in cereals [169]. Baking is another crucial process in the preparation of cereal-based meals, such as cookies, loaves, and cakes. Milani, et al. [170] reported that employing active dried or compressed yeast during the bread-making procedure lowered the amounts of AFs in bread by 32–64%. Further, in one of the studies involving muffins prepared from corn originally contaminated with AFB1, the toxin was reduced from 87 to ± 4% due to the frying process [171]. Another approach for lowering AF concentrations is nixtamalization (alkalization at higher temperatures), which is employed in manufacturing corn chips, tortilla chips, and corn tortillas. This treatment resulted in a 51–78% reduction in AF concentrations for various AF subtypes [172].

Plasma is the fourth state of matter and an emerging technology that shows great potential in various applications. Plasma is a quasi-neutral ionized gas that is primarily composed of free electrons, photons, and ions. Based on its generation, it can be classified as thermal and non-thermal plasma. Treatment with cold atmospheric plasma significantly reduces the microbial load without greatly affecting the quality of food. For example, plasma application at 40 W for 20 min significantly inhibited the growth of *A. flavus* for up to 20 days during storage at 25 °C [173]. Shi et al. [174] treated AF-contaminated corn with high-voltage cold atmospheric plasma under different conditions of the gas type, relative humidity, and treatment time. The results showed that the combination of MA65 gas, higher relative humidity (80%), and longer time (10 min) degraded the AF content up to 82%. However, the loss of dietary material and/or nutrients should be considered when lowering mycotoxin levels. Mycotoxin reduction treatments may also release and increase the bioavailability of masked mycotoxins in treated products [175] or alter their chemical structures into forms that are undetectable by standard analytical methods while preserving their toxicity [176]. Hence, specific analytical tools are required to discover structural alterations and links to the food matrix to solve these issues.

### 7.3. Chemical Methods

Chemical methods are generally easier to apply and quicker in their responses post-treatment. Mostly plant protection chemicals such as fungicides and insecticides are used [177]. Some food additives are used for the disinfection of aflatoxin in food materials. Shi et al. [178] used four food additives, viz., sodium bisulfite, sodium hypochlorite, citric acid, and ammonium persulfate, to reduce aflatoxin content from distillers wet grains (DWG) and condensed distillers soluble (CDS). These DWG and CDS are nutrient-rich byproducts of shelled maize. Out of four food additives, citric acid showed the most significant result. It removed about 65 and 80% AF content in DWG and CD, respectively, at a 2.5% concertation when heated at 90 ℃ for 60 min.

Chlorine is a strong oxidizing agent that is used as a sanitizing and cleaning agent for water, cereals, and horticultural commodities. This effectively degrades organic compounds via electron transfer. Samples contaminated with aflatoxin were collected from China and treated with chlorine gas. AFB1 was broken down into four compounds, and it was found to be non-toxic when tested on human embryo hepatocytes. The highest degradation achieved was up to 90%; however, the optimal degradation efficiency was about 79.6%. So, the application of chlorine was found to be a very effective and economically viable approach to detoxifying AFB1 in grains [179]. Besides this, ozone (O_3_) treatment was also found to be beneficial in reducing AFB1 in maize. The effect of treatment was noted to occur in a time- and concentration-dependent manner; however, the efficacy was higher in low-moisture maize. Ozone treatment (90 mg/L) successfully removed about 78.16 to 88.07% of AFB1 with 20 and 40 min of treatment [180]. Similarly, the application of O_3_ (60 μmol/mol) for 180 min completely inhibited the growth of *A. flavus*, and O_3_ also degraded up to 95% AF in wheat [181].

Besides these practices, synthetic fungicides have also been used to manage AFs. Several synthetic fungicides have been proven to control AFs produced by *Aspergillus* sp. In a study, Aleksić et al. [182] investigated the use of pyrimethanil to control the AFs produced by *Aspergillus niger*. They reported that pyrimethanil effectively retarded the appearance of fungal rot [182]. Lagogianni and Tsitsigiannis [177] studied the efficacy of several synthetic fungicides (azoxystrobin, boscalid, cyprodinil, fludioxonil, and cyprodinil + fludioxonil) on AFs produced by *A. flavus* in maize. They reported that cyprodinil was the most effective fungicide (EC_50_ < 0.05 µg/mL), followed by fludioxonil (EC_50_ < 0.11 µg/mL). The least effective was boscalid (EC_50_ 4.35–4.50 µg/mL). Moreover, they reported that in a 2-year field study, cyprodinil + fludioxonil showed an 83% reduction in AF contamination by *A. flavus* in maize [177]. Prochloraz, an azole fungicide, is more effective than tebuconazole in controlling the growth and production of aflatoxin according to *Mateo* et al. [183], who assessed the efficacy of azole-based fungicides (prochloraz, tebuconazole, and prochloraz + tebuconazole [2:1 *w*/*w*]) to manage the AFs produced by *A. flavus* in maize kernels. They reported that prochloraz was more effective, followed by prochloraz + tebuconazole (2:1) and tebuconazole. Ferrigo et al. [184] reported the higher efficacy of prothioconazole + tebuconazole in controlling the growth of AFs produced by *A. flavus*. In addition, Masiello et al. [185] investigated the efficacy of the succinate dehydrogenase inhibitor fungicides boscalid and isopyrazam in controlling AFs produced by *A. flavus*. Further, Magnoli et al. [186] studied the use of chlorpyrifos to control the growth of aflatoxin B_1_ produced by the *Aspergillus* section *Flavi* strain. These approaches, however, have numerous disadvantages. These fungicides are harmful and pollute the environment. The usage of synthetic fungicides might pose health concerns. These synthetic fungicides are carcinogenic to humans.

### 7.4. Biological Methods

Biological detoxification involves using fungi, bacteria, and actinomycetes to reduce or completely remove AFs in food products through either adsorption or enzymatic degradation [89]. Some strains of microorganisms and volatiles have been reported to have a positive response in the inhibition of AFs. The volatiles produced by *Bacillus megaterium* KU143 and *Pseudomonas protegens* AS15 noticeably repressed the growth, sporulation, and conidial germination of *A. flavus* under in vitro conditions, as well as fungal populations in rice grains during storage [187]. Further, Shetty et al. [188] observed the AFB1-binding ability of *Saccharomyces cerevisiae*. The highest binding efficacy was observed to be up to 53% during the exponential growth phase, and the efficacy was reduced when reaching the stationary phase. 

In addition, the effect of six biopesticides/biostimulants, viz., Botector^®^, Mycostop^®^, Serenade Max^®^, Trianum^®^, Vacciplant^®^, and zeolite, was evaluated by Lagogianni and Tsitsigiannis [189] under in vitro and field conditions. Mycostop^®^, Serenade Max^®^, Vacciplant^®^, and zeolite significantly inhibited the growth of *A. flavus* conidia production by 38.8–63.1% under lab conditions. However, Mycostop^®^ and Botector^®^ treatments exhibited significant decreases in disease incidences of 16.5 and 21.9%, respectively. Further, they also reduced AFB1 concentrations in maize kernels by 43.05 and 43.09%, respectively, when applied twice during the silk stage. The biological agents contained in Mycostop^®^ and Botector^®^ are *Streptomyces griseoviridis* and *Aureobasidium pullulans*, respectively [189]. Additionally, a pre-harvest spray treatment of *Trichoderma harzianum* T77 on the silks of sweetcorn plants under greenhouse and field conditions exhibited the inhibition of *A. flavus* infection. The author suggested that the combination of proper post-harvest management could reduce AF content [190]. Hruska et al. [191] applied a non-aflatoxigenic *A. flavus* biocontrol strain on maize, which suppressed the growth of aflatoxigenic *A. flavus* by up to 82%, and consequently, a smaller population, with up to 73% of suppression, was observed in AF content.

### 7.5. Use of Phytochemicals

Due to the harmful effects and risks associated with synthetic chemicals, the demand for natural, safer, and eco-friendly methods has increased. Several plant extracts, including essential oils (EOs), are suitable alternatives [192]. A screening study of plants such as *Psorospermum febrifugum*, Prosopis Africana, and *Curcuma longa* showed the presence of phytochemicals, including steroids, terpenoids, glycosides, phenols, tannins, saponins, and flavonoids, with anti-aflatoxigenic activity [193]. Kavitha et al. [194] reported that plants such as *Zingiber officinalis* (Zinger), *Oxalis corniculate* (Indian Sorrel), *Trigonella foenum-graecum* (Fenugreek), *Stevia rebaudiana* (Sugar Leaf), and *Equisetum arvense* (field horsetail) have anti-aflatoxigenic and anti-fungal activities. The aqueous leaf extract of *Salvia farinacea* and *Azadirachta indica* at 2 mg/mL has been reported to have antifungal activity against *Aspergillus *parasiticus** in different food samples. Phytochemicals extracted from *Thymus* spp. have antifungal activity against *A. flavus* and *A. parasiticus*. Thymol from *Thymus *kotschyanus** was used against *A. flavus* at 0.5 μg/mL [195]. Similarly, curcumin has the potential to inhibit *A.* *flavus* by inhibiting the Cyt450 isoenzyme CYP2A6 and reducing the formation of AFB1-8, 9 epoxide [196]. Several phytochemicals against AF-producing fungi are presented in Table 2.

Further, EOs have the ability to cross the plasma membrane and cause lipid partition in the cell membrane of fungi and the subsequent leakage of the cell contents. EOs can decrease the biosynthesis of compounds such as ergosterol and sterol [212]. EOs from six plant species (*Rosmarinus officinalis*, *Thymus vulgaris*, *Satureja montana*, *Origanum virens*, *O. majoricum*, and *O. vulgare*) were tested in in vitro conditions against *A. flavus* and AF contents. At the higher concentration, all six EOs showed great efficiency and completely inhibited fungal growth as well as inhibited about 100% of AFB1 production at 500 µg/mL [201]. EOs from anise (*Pimpinella anisum*) and boldus (*Peumus boldus*) were effective against *Aspergillus parasiticus* and *Aspergillus flavus* in stored maize [212,213]. EOs from bergamot, bitter orange, and lemon at 2% showed the inhibition of mycelial growth as well as AFB1 production by *A. flavus*. These EOs can be recovered and reused from citrus waste and are eco-friendly fungicides that could also be profitable for the agriculture sector [214]. The EOs extracted from *Cananga odorata* showed antifungal activity against *A. parasiticus* and *A. flavus* at 4 mg/mL [215]. The shelf life of brown rice was enhanced by *Michelia alba* oil in combination with linalool and caryophyllene at different ratios, while a 1:10 ratio showed the strongest antifungal activity against *A. flavus* [216]. EOs from the seeds of *Anethum graveolens* were reported to have inhibitory potential against *A. flavus*, along with other fungi at a MIC (minimum inhibitory concentration) of 120 µL/mL [217]. *A. flavus* sporulation on infected maize seeds was significantly suppressed by black *Piper nigrum* essential oil (BPEO) fumigation, with 100% inhibition being the highest result at 50 and 100 µL/mL of BPEO [218]. The nanoencapsulated *Pimpinella anisum* essential oil (PAEO) was found to preserve the stored rice samples against fungal and AFB1 contamination [219]. The nanoencapsulated EO of *Cananga odorata* with chitosan has been reported to have in vitro and in vivo preservative actions against *A. flavus* contamination and AFB1 production [220]. 

Furthermore, a phytochemical, terpinen-4-ol (28.92%) from *Origanum majorana* EO (nanoencapsulated), was reported to cause the *in situ* inhibition of lipid peroxidation and AFB1 production in maize [221]. In addition, ginger essential oils (GEO) containing 23.85% α-zingiberene and 14.16% geranial inhibited *A. flavus* growth and AFB1/AFB2 production, respectively, at 25 and 50 μg/g GEO in stored maize grains [222]. However, the efficacies of plant extracts and their phytochemicals in the management of toxigenic fungi and their toxins inherit limitations to solely be used as biofungicides and nutraceuticals [196]. Hence, these can be used in combination with the other detoxification methods discussed above for the better control and management of AFs in cereals and cereal-based products.

## 8. Conclusions

Aflatoxin (AF) contamination of cereals and cereal-based products has caused severe health concerns for humans, in addition to substantial economic losses. AF formation is influenced by various parameters in field conditions and/or during storage. Further, their detoxification using physical, chemical, and biological methods can be controlled and managed to some extent. The growing awareness of the adverse effects of synthetic chemicals used in conventional practices on human health and the environment has focused the interest of researchers on AF management using phytochemicals and their essential oils (EOs). In addition, the use of phytochemicals has proven to be a natural, safe, and eco-friendly method for AF detoxification, benefiting both the environment and the consumer. These practices are sustainable, as they are natural and environmentally friendly. Thus, they pose no risk to the environment or consumers. These phytochemicals release some EOs that are effective against AFs. These EOs may be reused and recovered, making them more environmentally friendly and sustainable. Thus, this practice for AF detoxification ensures food safety, as these agents are free from any toxic residues that are generated due to employing conventional practices. Moreover, combining these phytochemicals with encapsulation techniques can detoxify AFs with little consequences. This approach further enhances the bioavailability of phytochemicals with high efficacy at low concentrations with strong AF-inhibiting properties. Furthermore, these phytochemicals play dual pharmacological and nutraceutical roles as biofungicides and detoxifying agents in mitigating the effects of AFs. Thus, phytochemicals can be used as an alternative AF detoxification practice to ensure food safety. To ensure food safety, there is a strict need for effective and safer management practices that can delay the growth of AFs in food without altering their sensorial attributes. Moreover, phytochemicals and their EOs have been regarded as GRAS (Generally Recognized as Safe). Using phytochemicals and their EOs strengthens their application in achieving “green consumerism” in the agriculture and food sector. However, further study is needed to examine the usefulness of phytochemicals in managing AFs. In-depth research on the interaction processes between encapsulated phytochemicals and food products and their impact on human and animal health should be conducted. Inadequate information in these areas highlights the need for more in-depth research into their chemical characteristics, biosynthetic pathways, and diverse practices for detoxification and management strategies to ensure food safety and security. 

## Figures and Tables

**Table 1:** Occurrence of aflatoxins in cereals and cereal-based products around the world.

Food Matrix	Country	No. of Samples	Aflatoxin	Range (μg/kg)	Limit of Detection (LOD, μg/kg)	Detection Technique	References
Barley-based products	Mediterranean area	1/4	AFB1	24	0.25	LC-MS/MS	[41]
Corn	Burkina Faso and Mozambique	13/26	AFB1	3.4–636	3.0	LC-MS/MS	[42]
	Burkina Faso and Mozambique	4/26	AFB2	7.4–46.3	6.0	LC-MS/MS	[42]
	India	28/150	AFB1	48–383	3.9	HPLC	[43]
	Tanzania	60	AFB1	3–1081	0.6	UPLC/TOFMS	[44]
	Tanzania	60	AFB2	12–177	0.4	UPLC/TOFMS	[44]
	Zimbabwe	95	AFB1	0–11	3.75	LC-MS/MS	[45]
	Zimbabwe	95	AFB1	0–3	1.75	LC-MS/MS	[45]
	Zimbabwe	80/388	AFB1	0.57–26.6	0.005	HPLC	[46]
	China	108	AFB1	0.4–136.8	0.1	HPLC	[47]
Corn ingredients	Costa Rica	108/970	Total AFs	0–290.4	0.01 & 3	ELISA and HPLC	[48]
Corn flour	Iran	30	AFB1	6.25–1060	2	UHPLC–MS/MS	[49]
Corn-based opaque beers	South Africa	2/32	AFB1	0–7	2.5	LC-MS	[50]
Corn	Africa	233	AFB1	19.2–1137.4	NA	ELISA	[51]
	Colombia	3/20	Total AFs	8.2–585.9	5	LC-MS/MS	[52]
	Turkey	38/1055	Total AFs	7.96–163.62	5	LC-MS/MS	[53]
	Ethiopia	NA	Total AFs	20–91.04	NA	HPLC	[54]
	Brazil	38/148	Total AFs	0.4–49.9	NA	LC-MS/MS	[55]
	South Korea	507	AFB1	1–5.2	0.1	LC/MS/MS	[56]
	Vietnam	1486/2370	AFB1	2–5	34.8	ELISA	[57]
	Niger and Benin	112	Total AFs	0–3000	NA	ELISA	[58]
	China	44	AFB1	0–148.4	1	HPLC	[59]
	Pakistan	72	Total AFs	0.5–10	0.5	HPLC	[60]
	Ghana	326	Total AFs	0–341	0.1	TLC	[61]
	Peru	82	Total AFs	1–17	0.4	LC-MS/MS	[62]
	Uganda	256	Total AFs	0–3760	NA	HPLC	[63]
	Togo	70	AFB1	1.1–75.9	0.08	HPLC	[64]
	Ghana	70/90	AFB1	0.78–339.3	0.13	HPLC	[65]
Corn flour	Serbia	27/56	Total AFs	1–9.14	0.4	HPLC-FD	[66]
Corn flour	Turkey	24	AFB1	0.041–1.12	0.026	HPLC	[67]
Corn bran	Uganda	40	Total AFs	7.5–393.5	1	HPLC	[68]
Corn bran	Tanzania	340	Total AFs	9.4–16.8	NA	ELISA	[69]
Pearl millet	South Korea	507	AFB1	1–1.1	0.1	LC/MS/MS	[56]
Pearl millet	Kenya	86	AFB1	0.4–5.6	NA	ELISA	[51]
Pearl millet	Tunisia	220	AFB1	117–1046	0.24	LC-MS/MS	[70]
Pearl millet	Tunisia	220	AFB2	0–96.1	0.40	LC-MS/MS	[70]
Rice	China	29	AFB1	0.1–1.4	0.1	HPLC	[47]
	Pakistan	88/120	Total AFs	1.18–11.46	0.4	TLC	[71]
	Pakistan	100/120	Total AFs	0.21–11.89	0.142	HPLC	[71]
	Pakistan	104/120	Total AFs	0.10–12.39	0.092	LC–MS/MS	[71]
	Pakistan	88/120	Total AFs	1.24–11.68	1.0	ELISA	[71]
	Nigeria	38	AFB1	3.7–20.2	0.15	LC-MS/MS	[72]
	Nigeria	38	AFB2	1.62–6.11	0.2	LC-MS/MS	[72]
	Nigeria	38	AFG1	3.76–7.21	0.2	LC-MS/MS	[72]
	Iran	40	AFB1	0.29–2.92	NA	ELISA	[73]
	China	235/370	AFB1	0.03–20	0.65	HPLC	[74]
	China	235/370	AFB1	0.0–1.6	0.15	HPLC	[74]
	Pakistan	2047	AFB1	1.17–6.91	1	TLC	[75]
	Bangladesh	227	AFB1	0–0.9	0.2	HPLC	[76]
	Thailand	240	AFB1	1.43–26.61	0.093	HPLC-FD	[77]
	India	2/87	Total AFs	21.58–22.98	NA	TLC	[78]
	Egypt	51	AFB1	100–200	NA	TLC	[79]
	Colombia	3/24	Total AFs	0.2–23.9	5	LC-MS/MS	[52]
	South Korea	507	AFB1	1–1.1	0.1	LC/MS/MS	[56]
	Mediterranean area	2/100	AFB1	26.0–33.0	0.25	LC-MS/MS	[41]
	Mediterranean area	1/100	AFB2	7.5	1.5	LC-MS/MS	[41]
Rice flour	Serbia	2/6	Total AFs	1.59–4.76	0.4	HPLC-FD	[66]
Rice flour	Turkey	16	AFB1	0–0.029	0.026	HPLC	[67]
Rice-based baby foods	Iran	27/30	AFB1	0–15.15	0.025	HPLC-FD	[80]
Sorghum	Africa	53	AFB1	11.9–23.1	NA	ELISA	[51]
	Ethiopia	90	AFB1	0–33.10	0.01–0.03	ELISA	[81]
	Nigeria	19/35	Total AFs	0.96-21.74	1	TLC	[82]
	India	15/21	AFB1	0.005–0.02	NA	TLC	[83]
	India	3/21	AFB2	0–0.005	NA	TLC	[83]
	South Korea	507	AFB1	0.7–1.7	0.1	LC/MS/MS	[56]
Sorghum malt (Omalodu)	Namibia	45	AFB1	0.61–28.3	0.17	LC/MS/MS	[84]
	Namibia	45	AFB2	0.14–2.35	0.04	LC/MS/MS	[84]
	Namibia	45	AFG1	0.39–6.95	0.1	LC/MS/MS	[84]
	Burkina Faso	20	AFB1	46.33–254.73	0.2	HPLC	[85]
Sorghum malt (Otambo)	Namibia	45	AFB1	0.56–54.2	0.17	LC/MS/MS	[84]
	Namibia	45	AFB2	0.5–4.48	0.04	LC/MS/MS	[84]
Sorghum beer	Namibia	45	AFG1	0.4	0.1	LC/MS/MS	[84]
Sorghum-based products	Mediterranean area	1/4	AFB1	0–6.4	0.25	LC-MS/MS	[41]
Wheat	Brazil	35	Total AFs	0–6.2	5.0	HPLC-FD	[86]
	Spain	14/60	AFB1	1.03–9.50	0.08	LC-MS/MS	[87]
	Spain	19/60	AFB2	0.34–0.67	0.08	LC-MS/MS	[87]
	Spain	6/60	AFG1	0.53–1.05	0.16	LC-MS/MS	[87]
	China	21/32	AFB1	0.03–0.12	0.03-0.2	LC-MS/MS	[88]
	Egypt	36	AFB1	0.13–49.79	0.04	HPLC	[89]
	Egypt	36	AFB2	0.09–2.96	0.12	HPLC	[89]
	Turkey	141	Total AFs	0.21–0.44	0.026	HPLC-FD	[90]
	Iran	4/16	AFB1	0–1.8	3	HPLC-FD	[80]
	Bangladesh	227	AFB1	0.9–1.6	0.2	HPLC	[76]
	Mediterranean area	3/21	AFB2	6.7–26.0	1.50	LC-MS/MS	[41]
Wheat-based products	Mediterranean area	10/65	AFB1	5.5–66.7	0.25	LC-MS/MS	[41]
	Mediterranean area	2/65	AFB2	5.6–7.6	1.5	LC-MS/MS	[41]
Wheat-based baby foods	Iran	4/16	AFB1	0–1.8	3	HPLC-FD	[80]
Multigrain-cereal baby foods	Iran	2/2	AFB1	1.03–2.50	3	HPLC-FD	[80]
Wheat bran	Brazil	32	Total AFs	4.8	5.0	HPLC-FD	[86]
Wheat flour	Iran	144/180	Total AFs	0.01–0.5	0.003	HPLC	[91]
Wheat flour	Turkey	60	AFB1	0–0.044	0.026	HPLC	[67]
Wheat flour	China	108	AFB1	0.1–0.9	0.1	HPLC	[47]
Wheat flour (whole)	Brazil	16	Total AFs	3.4	5.0	HPLC-FD	[86]
Wheat flour (refined)	Brazil	15	Total AFs	1.2	5.0	HPLC-FD	[86]
Wheat bran	Iran	54/60	Total AFs	0.06–0.99	0.01	HPLC	[91]

HPLC-FLD: high-performance liquid chromatography–fluorescence detector; DLLME-HPLC: dispersive liquid–liquid microextraction coupled to high-performance liquid chromatography with fluorescence detection; TLC: thin-layer chromatography; UHPLC/TOFMS: ultrahigh-performance liquid chromatography/time-of-flight mass spectrometry; ELISA: enzyme-linked immunosorbent assay; LC-MS/MS: liquid chromatography–tandem mass spectrometry; AFB1: aflatoxin B1; AFB2: aflatoxin B2; Total AFs: aflatoxins (B1 + B2 + G1 + G2).

**Table 2:** Phytochemicals from various plant sources effective against aflatoxin-producing fungi.

Phytochemical Source	Phytochemical Form	Target Fungi	Food Commodity	Outcomes	References
*Schinus mole* (Pepperina)	Nanoparticles	*A. parasiticus*	Maize	59% control of aflatoxin production;	[197]
*Rosmarinus officinalis* (Rosemary)	EOs	*Aspergillus flavus*	Not available (NA)	fungal contamination and production of AFB1 and AFB2 inhibited at 250 μL/mL	[198]
*Pelargonium* *graveolens* (Sweet scented or rose Scented Geranium)	Nanogel	*A. flavus*	Maize	77.96% prevention at 1.0 μL/mL of nanogel	[199]
*Carum carvi* (caraway), *Juniperus communis* (juniper)	EOs	*A. flavus*, *A. parasiticus*	Maize flour	Significant prevention of fungal contamination and aflatoxin production	[200]
Satureja Montana (winter savory), Origanum virens (Oregano)	Niosome	*A. flavus*	Maize	Reduction in fungal growth and aflatoxin accumulation	[201]
*Zataria multiflora* (Satar)	Solid lipid nanoparticles and EOs	*A. flavus*	NA	Enhanced antifungal activity observed	[202]
Satureja montana (winter savory), Origanum virens (Oregano)	EOs	*A. flavus*	NA	Significant reduction at 0.96aw	[203]
*Origanum vulgare* (Oregano), *Thymus vulgaris* (garden thyme), *Melaleuca* *alternifolia* (tea tree), *Mentha piperita* (Peppermint)	Nanocomposite films	*A. flavus*, *A. parasiticus*	Rice	51–77% reduction in fungal growth during storage	[204]
*Myristica fragrans* (Nutmeg)	Nanoemulsion	*A. flavus*	Rice	Significant inhibition of AFB1 production	[205]
Clove & Quercetin from *Syzygium aromaticum*	Phytochemical	*A. flavus*, *A. parasiticus*	NA	Inhibited AF production	[196]
Cyanidin from *Solanum lycopersicum*	Phytochemical	*A. flavus*, *A. parasiticus*	NA	Inhibition of AFB1 production	[206]
Curcumin from *Curcuma longa* L. (Turmeric)	Phytochemical	*A. flavus*	NA	Prevention of hyphae production	[207]
Turmeric EO (e.g. β-pinene, camphor, and eucalyptol)	EOs	*A. flavus*	NA	Fungicidal activity	[208]
Eugenyl acetate, eugenol, and β-caryophyllene from *Syzygium aromaticum*	EOs	*A. flavus*	NA	Caused apoptosis in fungal hyphae	[209]
*Brassica alba*, *Brassica juncea*, *Brassica nigra*	Allyl isothiocyanate	NA	NA	Antifungal activity	[210]
*Brassica nigra*	EOs	*A. fumigatus*, *A. nomius*, *A.niger*	NA	0.012–0.06 µg/mL inhibition determined by using vapor diffusion method	[211]
*Brassica nigra*	EOs	*A. niger*, *A. flavus*, *A. ochraceus*	-	0.8–50 µg/mL inhibition found using broth macrodilution method	[211]

## Data Availability

Not applicable.

## References

[1] Pankaj S.K., Shi H., Keener K.M. (2018). A review of novel physical and chemical decontamination technologies for aflatoxin in food. Trends Food Sci. Technol..

[2] Mahato D.K., Lee K.E., Kamle M., Devi S., Dewangan K.N., Kumar P., Kang S.G. (2019). Aflatoxins in food and feed: An overview on prevalence, detection and control strategies. Front. Microbiol..

[3] Kamle M., Mahato D.K., Devi S., Lee K.E., Kang S.G., Kumar P. (2019). Fumonisins: Impact on agriculture, food, and human health and their management strategies. Toxins.

[4] Kamle M., Mahato D.K., Gupta A., Pandhi S., Sharma B., Dhawan K., Mishra S., Kumar M., Tripathi A.D., Rasane P. (2022). Deoxynivalenol: An Overview on Occurrence, Chemistry, Biosynthesis, Health Effects and Its Detection, Management, and Control Strategies in Food and Feed. Microbiol. Res..

[5] Kamle M., Mahato D.K., Gupta A., Pandhi S., Sharma N., Sharma B., Mishra S., Arora S., Selvakumar R., Saurabh V. (2022). Citrinin Mycotoxin Contamination in Food and Feed: Impact on Agriculture, Human Health, and Detection and Management Strategies. Toxins.

[6] Campagnollo F.B., Ganev K.C., Khaneghah A.M., Portela J.B., Cruz A.G., Granato D., Corassin C.H., Oliveira C.A.F., Sant’Ana A.S. (2016). The occurrence and effect of unit operations for dairy products processing on the fate of aflatoxin M1: A review. Food Control.

[7] Pereira V.L., Fernandes J.O., Cunha S.C. (2014). Mycotoxins in cereals and related foodstuffs: A review on occurrence and recent methods of analysis. Trends Food Sci. Technol..

[8] Majeed M., Khaneghah A.M., Kadmi Y., Khan M.U., Shariati M.A. (2018). Assessment of ochratoxin A in commercial corn and wheat products. Curr. Nutr. Food Sci..

[9] Mahato D.K., Devi S., Pandhi S., Sharma B., Maurya K.K., Mishra S., Dhawan K., Selvakumar R., Kamle M., Mishra A.K. (2021). Occurrence, impact on agriculture, human health, and management strategies of zearalenone in food and feed: A review. Toxins.

[10] Mahato D.K., Kamle M., Sharma B., Pandhi S., Devi S., Dhawan K., Selvakumar R., Mishra D., Kumar A., Arora S. (2021). Patulin in food: A mycotoxin concern for human health and its management strategies. Toxicon.

[11] Kumar P., Mahato D.K., Sharma B., Borah R., Haque S., Mahmud M.M.C., Shah A.K., Rawal D., Bora H., Bui S. (2020). Ochratoxins in food and feed: Occurrence and its impact on human health and management strategies. Toxicon.

[12] Kumar P., Mahato D.K., Kamle M., Mohanta T.K., Kang S.G. (2017). Aflatoxins: A global concern for food safety, human health and their management. Front. Microbiol..

[13] Kumar P., Mahato D.K., Gupta A., Pandey S., Paul V., Saurabh V., Pandey A.K., Selvakumar R., Barua S., Kapri M. (2022). Nivalenol Mycotoxin Concerns in Foods: An Overview on Occurrence, Impact on Human and Animal Health and Its Detection and Management Strategies. Toxins.

[14] Mahato D.K., Pandhi S., Kamle M., Gupta A., Sharma B., Panda B.K., Srivastava S., Kumar M., Selvakumar R., Pandey A.K. (2022). Trichothecenes in food and feed: Occurrence, impact on human health and their detection and management strategies. Toxicon.

[15] Khaneghah A.M., Fakhri Y., Raeisi S., Armoon B., Sant’Ana A.S. (2018). Prevalence and concentration of ochratoxin A, zearalenone, deoxynivalenol and total aflatoxin in cereal-based products: A systematic review and meta-analysis. Food Chem. Toxicol..

[16] Jiang Y.I., Jolly P.E., Ellis W.O., Wang J.-S., Phillips T.D., Williams J.H. (2005). Aflatoxin B1 albumin adduct levels and cellular immune status in Ghanaians. Int. Immunol..

[17] Somorin Y.M., Bertuzzi T., Battilani P., Pietri A. (2012). Aflatoxin and fumonisin contamination of yam flour from markets in Nigeria. Food Control.

[18] Omara T., Kiprop A.K., Wangila P., Wacoo A.P., Kagoya S., Nteziyaremye P., Peter Odero M., Kiwanuka Nakiguli C., Baker Obakiro S. (2021). The scourge of aflatoxins in Kenya: A 60-year review (1960 to 2020). J. Food Qual..

[19] EFSA (2013). European Food Safety Authority. Aflatoxins (Sum of B1, B2, G1, G2) in Cereals and Cereal-Derived Food Products.

[20] Shabeer S., Asad S., Jamal A., Ali A. (2022). Aflatoxin Contamination, Its Impact and Management Strategies: An Updated Review. Toxins.

[21] Khaneghah A.M., Martins L.M., von Hertwig A.M., Bertoldo R., Sant’Ana A.S. (2018). Deoxynivalenol and its masked forms: Characteristics, incidence, control and fate during wheat and wheat based products processing—A review. Trends Food Sci. Technol..

[22] Andrade P.D., Caldas E.D. (2015). Aflatoxins in cereals: Worldwide occurrence and dietary risk assessment. World Mycotoxin J..

[23] WHO (2015). WHO Estimates of the Global Burden of Foodborne Diseases: Foodborne Disease Burden Epidemiology Reference Group 2007–2015.

[24] Ostry V., Malir F., Toman J., Grosse Y. (2017). Mycotoxins as human carcinogens—The IARC Monographs classification. Mycotoxin Res..

[25] Bhatnagar-Mathur P., Sunkara S., Bhatnagar-Panwar M., Waliyar F., Sharma K.K. (2015). Biotechnological advances for combating *Aspergillus flavus* and aflatoxin contamination in crops. Plant Sci..

[26] EC (2007). European Commission. Commission Regulation (EC) No1126/2007 of 28 September 2007 amending regulation (EC) no 1881/2006 setting maximum levels for certain contaminants in foodstuffs as regards *Fusarium* toxins in maize and maize products. Off. J. Eur. Union..

[27] EC (2010). European Commission. Commission Regulation (EC) No 165/2010 of 26 February 2010 amending regulation (EC) no 1881/2006 setting maximum levels for certain contaminants in foodstuffs as regards Fusarium toxins in maize and maize products. Off. J. Eur. Union..

[28] Wu F. (2006). Mycotoxin reduction in Bt corn: Potential economic, health, and regulatory impacts. Transgenic Res..

[29] Oliveira M., Pereira C., Bessa C., Araujo R., Saraiva L. (2015). Chronological aging in conidia of pathogenic *Aspergillus*: Comparison between species. J. Microbiol. Methods.

[30] Battilani P., Formenti S., Ramponi C., Rossi V. (2011). Dynamic of water activity in maize hybrids is crucial for fumonisin contamination in kernels. J. Cereal Sci..

[31] Negash D. (2018). A review of aflatoxin: Occurrence, prevention, and gaps in both food and feed safety. J. Nutr. Health Food Eng..

[32] Gnonlonfin G.J.B., Hell K., Adjovi Y., Fandohan P., Koudande D.O., Mensah G.A., Sanni A., Brimer L. (2013). A review on aflatoxin contamination and its implications in the developing world: A sub-Saharan African perspective. Crit. Rev. Food Sci. Nutr..

[33] Filazi A., Sireli U.T. (2013). Occurrence of aflatoxins in food. Aflatoxins: Recent Advances Future Prospects.

[34] Al-Zoreky N.S., Saleh F.A. (2019). Limited survey on aflatoxin contamination in rice. Saudi J. Biol. Sci..

[35] Achaglinkame M.A., Opoku N., Amagloh F.K. (2017). Aflatoxin contamination in cereals and legumes to reconsider usage as complementary food ingredients for Ghanaian infants: A review. J. Nutr. Intermed. Metab..

[36] Schmidt-Heydt M., Rüfer C.E., Abdel-Hadi A., Magan N., Geisen R. (2010). The production of aflatoxin B 1 or G 1 by *Aspergillus parasiticus* at various combinations of temperature and water activity is related to the ratio of aflS to afl R expression. Mycotoxin Res..

[37] Lv C., Jin J., Wang P., Dai X., Liu Y., Zheng M., Xing F. (2019). Interaction of water activity and temperature on the growth, gene expression and aflatoxin production by *Aspergillus flavus* on paddy and polished rice. Food Chem..

[38] Gizachew D., Chang C.-H., Szonyi B., De La Torre S., Ting W.-t.E. (2019). Aflatoxin B1 (AFB1) production by *Aspergillus flavus* and *Aspergillus parasiticus* on ground Nyjer seeds: The effect of water activity and temperature. Int. J. Food Microbiol..

[39] Battilani P., Toscano P., Van der Fels-Klerx H.J., Moretti A., Leggieri M.C., Brera C., Rortais A., Goumperis T., Robinson T. (2016). Aflatoxin B 1 contamination in maize in Europe increases due to climate change. Sci. Rep..

[40] Moretti A., Pascale M., Logrieco A.F. (2019). Mycotoxin risks under a climate change scenario in Europe. Trends Food Sci. Technol..

[41] Serrano A.B., Font G., Ruiz M.J., Ferrer E. (2012). Co-occurrence and risk assessment of mycotoxins in food and diet from Mediterranean area. Food Chem..

[42] Warth B., Parich A., Atehnkeng J., Bandyopadhyay R., Schuhmacher R., Sulyok M., Krska R. (2012). Quantitation of mycotoxins in food and feed from Burkina Faso and Mozambique using a modern LC-MS/MS multitoxin method. J. Agric. Food Chem..

[43] Mudili V., Siddaih C.N., Nagesh M., Garapati P., Naveen Kumar K., Murali H.S., Yli Mattila T., Batra H.V. (2014). Mould incidence and mycotoxin contamination in freshly harvested maize kernels originated from India. J. Sci. Food Agric..

[44] Kamala A., Ortiz J., Kimanya M., Haesaert G., Donoso S., Tiisekwa B., De Meulenaer B. (2015). Multiple mycotoxin co-occurrence in maize grown in three agro-ecological zones of Tanzania. Food Control.

[45] Hove M., De Boevre M., Lachat C., Jacxsens L., Nyanga L., De Saeger S. (2016). Occurrence and risk assessment of mycotoxins in subsistence farmed maize from Zimbabwe. Food Control.

[46] Murashiki T.C., Chidewe C., Benhura M.A., Maringe D.T., Dembedza M.P., Manema L.R., Mvumi B.M., Nyanga L.K. (2017). Levels and daily intake estimates of aflatoxin B1 and fumonisin B1 in maize consumed by rural households in Shamva and Makoni districts of Zimbabwe. Food Control.

[47] Sun G., Wang S., Hu X., Su J., Zhang Y., Xie Y., Zhang H., Tang L., Wang J.-S. (2011). Co-contamination of aflatoxin B1 and fumonisin B1 in food and human dietary exposure in three areas of China. Food Addit. Contam..

[48] Granados-Chinchilla F., Molina A., Chavarría G., Alfaro-Cascante M., Bogantes-Ledezma D., Murillo-Williams A. (2017). Aflatoxins occurrence through the food chain in Costa Rica: Applying the One Health approach to mycotoxin surveillance. Food Control.

[49] Amirahmadi M., Shoeibi S., Rastegar H., Elmi M., Mousavi Khaneghah A. (2018). Simultaneous analysis of mycotoxins in corn flour using LC/MS-MS combined with a modified QuEChERS procedure. Toxin Rev..

[50] Adekoya I., Obadina A., Adaku C.C., De Boevre M., Okoth S., De Saeger S., Njobeh P. (2018). Mycobiota and co-occurrence of mycotoxins in South African maize-based opaque beer. Int. J. Food Microbiol..

[51] Sirma A.J., Senerwa D.M., Grace D., Makita K., Mtimet N., Kang’ethe E.K., Lindahl J.F. (2016). Aflatoxin B1 occurrence in millet, sorghum and maize from four agro-ecological zones in Kenya. Afr. J. Food Agric. Nutr. Dev..

[52] Diaz G.J., Krska R., Sulyok M. (2015). Mycotoxins and cyanogenic glycosides in staple foods of three indigenous people of the Colombian Amazon. Food Addit. Contam. Part B.

[53] Artik N., Konar N., Özkan M., Çakmakçi M.L. (2016). Aflatoxin and genetically modified organisms analysis in Turkish corn. Food Sci. Nutr..

[54] Chauhan N.M. (2017). Aflatoxin: A Risky Menace for African’s Food Commodities. Aflatoxin: Control, Analysis, Detection Health Risks.

[55] Oliveira M.S., Rocha A., Sulyok M., Krska R., Mallmann C.A. (2017). Natural mycotoxin contamination of maize (*Zea mays* L.) in the South region of Brazil. Food Control.

[56] Kim D.-H., Hong S.-Y., Kang J.W., Cho S.M., Lee K.R., An T.K., Lee C., Chung S.H. (2017). Simultaneous determination of multi-mycotoxins in cereal grains collected from South Korea by LC/MS/MS. Toxins.

[57] Lee H.S., Nguyen-Viet H., Lindahl J., Thanh H.M., Khanh T.N., Hien L.T.T., Grace D. (2017). A survey of aflatoxin B1 in maize and awareness of aflatoxins in Vietnam. World Mycotoxin J..

[58] Bakoye O.N., Baoua I.B., Seyni H., Amadou L., Murdock L.L., Baributsa D. (2017). Quality of maize for sale in markets in Benin and Niger. J. Stored Prod. Res..

[59] Xing F., Liu X., Wang L., Selvaraj J.N., Jin N., Wang Y., Zhao Y., Liu Y. (2017). Distribution and variation of fungi and major mycotoxins in pre-and post-nature drying maize in North China Plain. Food Control.

[60] Manzoor M., Farooq Z., Iqbal S., Mukhtar H., Nawaz M. (2018). Quantification of aflatoxins in maize samples collected from various parts of the Punjab, Pakistan. J. Anim. Plant Sci..

[61] Agbetiameh D., Ortega-Beltran A., Awuah R.T., Atehnkeng J., Cotty P.J., Bandyopadhyay R. (2018). Prevalence of aflatoxin contamination in maize and groundnut in Ghana: Population structure, distribution, and toxigenicity of the causal agents. Plant Dis..

[62] Coloma Z.N., Oliveira M.S., Dilkin P., Mallmann A.O., Almeida C.A.A., Mallmann C.A. (2019). Mycotoxin occurrence in Peruvian purple maize. World Mycotoxin J..

[63] Sserumaga J.P., Ortega-Beltran A., Wagacha J.M., Mutegi C.K., Bandyopadhyay R. (2020). Aflatoxin-producing fungi associated with pre-harvest maize contamination in Uganda. Int. J. Food Microbiol..

[64] Hanvi M.D., Lawson-Evi P., Bouka E.C., Eklu-Gadegbeku K. (2021). Aflatoxins in maize dough and dietary exposure in rural populations of Togo. Food Control.

[65] Kortei N.K., Annan T., Akonor P.T., Richard S.A., Annan H.A., Kyei-Baffour V., Akuamoa F., Akpaloo P.G., Esua-Amoafo P. (2021). The occurrence of aflatoxins and human health risk estimations in randomly obtained maize from some markets in Ghana. Sci. Rep..

[66] Torović L. (2018). Aflatoxins and ochratoxin A in flour: A survey of the Serbian retail market. Food Addit. Contam. Part B.

[67] Kara G.N., Ozbey F., Kabak B. (2015). Co-occurrence of aflatoxins and ochratoxin A in cereal flours commercialised in Turkey. Food Control.

[68] Nakavuma J.L., Kirabo A., Bogere P., Nabulime M.M., Kaaya A.N., Gnonlonfin B. (2020). Awareness of mycotoxins and occurrence of aflatoxins in poultry feeds and feed ingredients in selected regions of Uganda. Int. J. Food Contam..

[69] Kajuna F.F., Temba B.A., Mosha R.D. (2013). Surveillance of aflatoxin B1 contamination in chicken commercial feeds in Morogoro, Tanzania. Livest. Res. Rural. Dev..

[70] Houissa H., Lasram S., Sulyok M., Šarkanj B., Fontana A., Strub C., Krska R., Schorr-Galindo S., Ghorbel A. (2019). Multimycotoxin LC-MS/MS analysis in pearl millet (*Pennisetum glaucum*) from Tunisia. Food Control.

[71] Iqbal J., Asghar M.A., Ahmed A., Khan M.A., Jamil K. (2014). Aflatoxins contamination in Pakistani brown rice: A comparison of TLC, HPLC, LC–MS/MS and ELISA techniques. Toxicol. Mech. Methods.

[72] Rofiat A.-S., Fanelli F., Atanda O., Sulyok M., Cozzi G., Bavaro S., Krska R., Logrieco A.F., Ezekiel C.N. (2015). Fungal and bacterial metabolites associated with natural contamination of locally processed rice (*Oryza sativa* L.) in Nigeria. Food Addit. Contam. Part A.

[73] Eslami M., Mashak Z., Heshmati A., Shokrzadeh M., Mozaffari Nejad A.S. (2015). Determination of aflatoxin B1 levels in Iranian rice by ELISA method. Toxin Rev..

[74] Lai X., Liu R., Ruan C., Zhang H., Liu C. (2015). Occurrence of aflatoxins and ochratoxin A in rice samples from six provinces in China. Food Control.

[75] Asghar M.A., Iqbal J., Ahmed A., Shamsuddin Z.A., Khan M.A. (2016). Incidence of aflatoxins in export quality basmati rice collected from different areas of Pakistan. Sci. Technol. Dev..

[76] Roy M., Harris J., Afreen S., Deak E., Gade L., Balajee S.A., Park B., Chiller T., Luby S. (2013). Aflatoxin contamination in food commodities in Bangladesh. Food Addit. Contam. Part B.

[77] Panrapee I., Phakpoom K., Thanapoom M., Nampeung A., Warapa M. (2016). Exposure to aflatoxin B 1 in Thailand by consumption of brown and color rice. Mycotoxin Res..

[78] Mukherjee A., Sharma M., Latkar S.S. (2019). A study on Aflatoxin content in black scented rice in India. Int. J. Pharm. Anal. Res..

[79] Moharram A.M., Yasser M.M., Sayed M.A., Omar O.A., Idres M.M.M. (2019). Mycobiota and mycotoxins contaminating rice grains in El-Minia, Governorate, Egypt. Biosci. Biotechnol. Res. Asia.

[80] Mottaghianpour E., Nazari F., Mehrasbi M.R., Hosseini M.J. (2017). Occurrence of aflatoxin B1 in baby foods marketed in Iran. J. Sci. Food Agric..

[81] Taye W., Ayalew A., Chala A., Dejene M. (2016). Aflatoxin B1 and total fumonisin contamination and their producing fungi in fresh and stored sorghum grain in East Hararghe, Ethiopia. Food Addit. Contam. Part B.

[82] Apeh D.O., Ochai D.O., Adejumo A., Muhammad H.L., Saidu A.N., Atehnkeng J., Adeyemi R.H., Mailafiya S.C., Makun H.A. (2016). Mycotoxicological concerns with sorghum, millet and sesame in Northern Nigeria. J. Anal. Bioanal. Technol..

[83] Jayashree M., Wesely E. (2019). Effect of moisture content on aflatoxin production in field infected and farmer saved sorghum (FSS) grains. Int. J. Anal. Exp. Modal Anal..

[84] Nafuka S.N., Misihairabgwi J.M., Bock R., Ishola A., Sulyok M., Krska R. (2019). Variation of fungal metabolites in sorghum malts used to prepare Namibian traditional fermented beverages Omalodu and Otombo. Toxins.

[85] Bationo J.F., Nikiéma P.A., Koudougou K., Ouédraogo M., Bazié S.R., Sanou E., Barro N. (2015). Assessment of aflatoxin B1 and ochratoxin A levels in sorghum malts and beer in Ouagadougou. Afr. J. Food Sci..

[86] Trombete F.M., de Ávila Moraes D., Porto Y.D., Santos T.B., Direito G.M., Fraga M.E., Saldanha T. (2014). Determination of aflatoxins in wheat and wheat by-products intended for human consumption, marketed in Rio de Janeiro, Brazil. J. Food Nutr. Res..

[87] Quiles J.M., Saladino F., Mañes J., Fernández-Franzón M., Meca G. (2016). Occurrence of mycotoxins in refrigerated pizza dough and risk assessment of exposure for the Spanish population. Food Chem. Toxicol..

[88] Zhao Y., Wang Q., Huang J., Ma L., Chen Z., Wang F. (2018). Aflatoxin B1 and sterigmatocystin in wheat and wheat products from supermarkets in China. Food Addit. Contam. Part B.

[89] Hathout A.S., Abel-Fattah S.M., Abou-Sree Y.H., Fouzy A.S.M. (2020). Incidence and exposure assessment of aflatoxins and ochratoxin A in Egyptian wheat. Toxicol. Rep..

[90] Turksoy S., Kabak B. (2020). Determination of aflatoxins and ochratoxin A in wheat from different regions of Turkey by HPLC with fluorescence detection. Acta Aliment..

[91] Jahanbakhsh M., Afshar A., Momeni Feeli S., Pabast M., Ebrahimi T., Mirzaei M., Akbari-Adergani B., Farid M., Arabameri M. (2021). Probabilistic health risk assessment (Monte Carlo simulation method) and prevalence of aflatoxin B1 in wheat flours of Iran. Int. J. Environ. Anal. Chem..

[92] Pavao A.C., Neto L.A.S., Neto J.F., Leao M.B.C. (1995). Structure and activity of aflatoxins B and G. J. Mol. Struct..

[93] Lalah J.O., Omwoma S., Orony D.A. (2019). Aflatoxin B1: Chemistry, environmental and diet sources and potential exposure in human in Kenya. Aflatoxin B1 Occurrence, Detection Toxicological Effects.

[94] Wogan G.N., Kensler T.W., Groopman J.D. (2012). Present and future directions of translational research on aflatoxin and hepatocellular carcinoma. A review. Food Addit. Contam. Part A.

[95] Amare M.G., Keller N.P. (2014). Molecular mechanisms of *Aspergillus flavus* secondary metabolism and development. Fungal Genet. Biol..

[96] Caceres I., Al Khoury A., El Khoury R., Lorber S., Oswald I.P., El Khoury A., Atoui A., Puel O., Bailly J.-D. (2020). Aflatoxin biosynthesis and genetic regulation: A review. Toxins.

[97] Crawford J.M., Vagstad A.L., Ehrlich K.C., Townsend C.A. (2008). Starter unit specificity directs genome mining of polyketide synthase pathways in fungi. Bioorg. Chem..

[98] Ehrlich K.C., Li P., Scharfenstein L., Chang P.-K. (2010). HypC, the anthrone oxidase involved in aflatoxin biosynthesis. Appl. Environ. Microbiol..

[99] Zhou R., Linz J.E. (1999). Enzymatic function of the Nor-1 protein in aflatoxin biosynthesis in *Aspergillus parasiticus*. Appl. Environ. Microbiol..

[100] Yu J., Bhatnagar D., Cleveland T.E. (2004). Completed sequence of aflatoxin pathway gene cluster in *Aspergillus parasiticus*. FEBS Lett..

[101] Chang P.-K., Yu J., Ehrlich K.C., Boue S.M., Montalbano B.G., Bhatnagar D., Cleveland T.E. (2000). adhA in *Aspergillus parasiticus* is involved in conversion of 5′-hydroxyaverantin to averufin. Appl. Environ. Microbiol..

[102] Sakuno E., Wen Y., Hatabayashi H., Arai H., Aoki C., Yabe K., Nakajima H. (2005). *Aspergillus parasiticus* cyclase catalyzes two dehydration steps in aflatoxin biosynthesis. Appl. Environ. Microbiol..

[103] Sakuno E., Yabe K., Nakajima H. (2003). Involvement of two cytosolic enzymes and a novel intermediate, 5′-oxoaverantin, in the pathway from 5′-hydroxyaverantin to averufin in aflatoxin biosynthesis. Appl. Environ. Microbiol..

[104] Wen Y., Hatabayashi H., Arai H., Kitamoto H.K., Yabe K. (2005). Function of the cypX and moxY genes in aflatoxin biosynthesis in *Aspergillus parasiticus*. Appl. Environ. Microbiol..

[105] Chang P.-K., Yabe K., Yu J. (2004). The *Aspergillus parasiticus* estA-encoded esterase converts versiconal hemiacetal acetate to versiconal and versiconol acetate to versiconol in aflatoxin biosynthesis. Appl. Environ. Microbiol..

[106] Lin B.-K., Anderson J.A. (1992). Purification and properties of versiconal cyclase from *Aspergillus parasiticus*. Arch. Biochem. Biophys..

[107] Ehrlich K.C., Montalbano B., Boué S.M., Bhatnagar D. (2005). An aflatoxin biosynthesis cluster gene encodes a novel oxidase required for conversion of versicolorin A to sterigmatocystin. Appl. Environ. Microbiol..

[108] Henry K.M., Townsend C.A. (2005). Ordering the reductive and cytochrome P450 oxidative steps in demethylsterigmatocystin formation yields general insights into the biosynthesis of aflatoxin and related fungal metabolites. J. Am. Chem. Soc..

[109] Yu J., Woloshuk C.P., Bhatnagar D., Cleveland T.E. (2000). Cloning and characterization of avfA and omtB genes involved in aflatoxin biosynthesis in three *Aspergillus* species. Gene.

[110] Yu J. (2012). Current understanding on aflatoxin biosynthesis and future perspective in reducing aflatoxin contamination. Toxins.

[111] Zeng H., Hatabayashi H., Nakagawa H., Cai J., Suzuki R., Sakuno E., Tanaka T., Ito Y., Ehrlich K.C., Nakajima H. (2011). Conversion of 11-hydroxy-O-methylsterigmatocystin to aflatoxin G 1 in *Aspergillus parasiticus*. Appl. Microbiol. Biotechnol..

[112] Nazhand A., Durazzo A., Lucarini M., Souto E.B., Santini A. (2020). Characteristics, occurrence, detection and detoxification of aflatoxins in foods and feeds. Foods.

[113] Georgianna D.R., Payne G.A. (2009). Genetic regulation of aflatoxin biosynthesis: From gene to genome. Fungal Genet. Biol..

[114] Chang P.K. (2003). The *Aspergillus parasiticus* protein AFLJ interacts with the aflatoxin pathway-specific regulator AFLR. Mol. Genet. Genom..

[115] Price M.S., Yu J., Nierman W.C., Kim H.S., Pritchard B., Jacobus C.A., Bhatnagar D., Cleveland T.E., Payne G.A. (2006). The aflatoxin pathway regulator AflR induces gene transcription inside and outside of the aflatoxin biosynthetic cluster. FEMS Microbiol. Lett..

[116] Ehrlich K.C., Chang P.-K., Yu J., Cotty P.J. (2004). Aflatoxin biosynthesis cluster gene cypA is required for G aflatoxin formation. Appl. Environ. Microbiol..

[117] Ehrlich K.C., Scharfenstein L.L., Montalbano B.G., Chang P.-K. (2008). Are the genes nadA and norB involved in formation of aflatoxin G1?. Int. J. Mol. Sci..

[118] Grace D., Mahuku G., Hoffmann V., Atherstone C., Upadhyaya H.D., Bandyopadhyay R. (2015). International agricultural research to reduce food risks: Case studies on aflatoxins. Food Secur..

[119] Gong Y.Y., Watson S., Routledge M.N. (2016). Aflatoxin exposure and associated human health effects, a review of epidemiological studies. Food Saf..

[120] Fouad A.M., Ruan D., El-Senousey H.K., Chen W., Jiang S., Zheng C. (2019). Harmful effects and control strategies of aflatoxin b1 produced by *Aspergillus flavus* and *Aspergillus parasiticus* strains on poultry. Toxins.

[121] Bou Zerdan M., Moussa S., Atoui A., Assi H.I. (2021). Mechanisms of immunotoxicity: Stressors and evaluators. Int. J. Mol. Sci..

[122] Benkerroum N. (2020). Chronic and acute toxicities of aflatoxins: Mechanisms of action. Int. J. Environ. Res. Public Health.

[123] Benkerroum N. (2019). Retrospective and prospective look at aflatoxin research and development from a practical standpoint. Int. J. Environ. Res. Public Health.

[124] Rushing B.R., Selim M.I. (2017). Structure and oxidation of pyrrole adducts formed between aflatoxin B2a and biological amines. Chem. Res. Toxicol..

[125] Zhuang Z., Huang Y., Yang Y., Wang S. (2016). Identification of AFB1-interacting proteins and interactions between RPSA and AFB1. J. Hazard. Mater..

[126] Klaunig J.E., Kamendulis L.M., Hocevar B.A. (2010). Oxidative stress and oxidative damage in carcinogenesis. Toxicol. Pathol..

[127] Ayala A., Muñoz M.F., Argüelles S. (2014). Lipid peroxidation: Production, metabolism, and signaling mechanisms of malondialdehyde and 4-hydroxy-2-nonenal. Oxid. Med. Cell. Longev..

[128] Oms-Oliu G., Martín-Belloso O., Soliva-Fortuny R. (2010). Pulsed light treatments for food preservation. A review. Food Bioprocess Technol..

[129] Castellari C.C., Cendoya M.G., FJ M.V., Barrera V., Pacin A.M. (2015). Extrinsic and intrinsic factors associated with mycotoxigenic fungi populations of maize grains (*Zea mays* L.) stored in silobags in Argentina. Rev. Argent. Microbiol..

[130] Schmidt-Heydt M., Magan N., Geisen R. (2008). Stress induction of mycotoxin biosynthesis genes by abiotic factors. FEMS Microbiol. Lett..

[131] Barbosa-Cánovas G.V., Fontana A.J., Schmidt S.J., Labuza T.P. (2020). Water Activity in Foods: Fundamentals and Applications.

[132] Jaime-Garcia R., Cotty P.J. (2003). Aflatoxin contamination of commercial cottonseed in south Texas. Phytopathology.

[133] Milani J.M. (2013). Ecological conditions affecting mycotoxin production in cereals: A review. Vet. Med..

[134] Pitt J.I., Miscamble B.F. (1995). Water relations of *Aspergillus flavus* and closely related species. J. Food Prot..

[135] Giorni P., Battilani P., Pietri A., Magan N. (2008). Effect of aw and CO2 level on *Aspergillus flavus* growth and aflatoxin production in high moisture maize post-harvest. Int. J. Food Microbiol..

[136] Fountain J.C., Scully B.T., Chen Z.-Y., Gold S.E., Glenn A.E., Abbas H.K., Lee R.D., Kemerait R.C., Guo B. (2015). Effects of hydrogen peroxide on different toxigenic and atoxigenic isolates of *Aspergillus flavus*. Toxins.

[137] Chulze S.N. (2010). Strategies to reduce mycotoxin levels in maize during storage: A review. Food Addit. Contam..

[138] Zhang K., Banerjee K. (2020). A review: Sample preparation and chromatographic technologies for detection of aflatoxins in foods. Toxins.

[139] Namjoo M., Salamat F., Rajabli N., Hajihoseeini R., Niknejad F., Kohsar F., Joshaghani H. (2016). Quantitative determination of aflatoxin by high performance liquid chromatography in wheat silos in Golestan province, north of Iran. Iran. J. Public Health.

[140] Mishra G., Panda B.K., Ramirez W.A., Jung H., Singh C.B., Lee S.H., Lee I. (2021). Research advancements in optical imaging and spectroscopic techniques for nondestructive detection of mold infection and mycotoxins in cereal grains and nuts. Compr. Rev. Food Sci. Food Saf..

[141] Zhang J., Xu B., Wang Z., Cheng F. (2021). Application of hyperspectral imaging in the detection of aflatoxin B1 on corn seed. J. Food Meas. Charact..

[142] Jia B., Wang W., Ni X.Z., Chu X., Yoon S.C., Lawrence K.C. (2020). Detection of mycotoxins and toxigenic fungi in cereal grains using vibrational spectroscopic techniques: A review. World Mycotoxin J..

[143] Liu S.-H., Wen B.-Y., Lin J.-S., Yang Z.-W., Luo S.-Y., Li J.-F. (2020). Rapid and Quantitative Detection of Aflatoxin B1 in Grain by Portable Raman Spectrometer. Appl. Spectrosc..

[144] Kasoju A., Shrikrishna N.S., Shahdeo D., Khan A.A., Alanazi A.M., Gandhi S. (2020). Microfluidic paper device for rapid detection of aflatoxin B1 using an aptamer based colorimetric assay. RSC Adv..

[145] Yadav N., Yadav S.S., Chhilar A.K., Rana J.S. (2021). An overview of nanomaterial based biosensors for detection of Aflatoxin B1 toxicity in foods. Food Chem. Toxicol..

[146] Xue Z., Zhang Y., Yu W., Zhang J., Wang J., Wan F., Kim Y., Liu Y., Kou X. (2019). Recent advances in aflatoxin B1 detection based on nanotechnology and nanomaterials—A review. Anal. Chim. Acta..

[147] Geleta G.S., Zhao Z., Wang Z. (2018). A novel reduced graphene oxide/molybdenum disulfide/polyaniline nanocomposite-based electrochemical aptasensor for detection of aflatoxin B 1. Analyst.

[148] Shkembi X., Svobodova M., Skouridou V., Bashammakh A.S., Alyoubui A.O. (2021). Aptasensors for mycotoxin detection: A review. Anal. Biochem..

[149] Renuka R.M., Achuth J., Mudili V., Poda S. (2018). Development of a FRET-based fluorescence aptasensor for the detection of aflatoxin B1 in contaminated food grain samples. RSC Adv..

[150] Mukherjee M., Bhatt P., HK M. (2017). Fluorescent competitive aptasensor for detection of aflatoxin B1. J. Mol. Recognit..

[151] Mahuku G., Nzioki H.S., Mutegi C., Kanampiu F., Narrod C., Makumbi D. (2019). Pre-harvest management is a critical practice for minimizing aflatoxin contamination of maize. Food Control.

[152] Brooks T.D., Williams W.P., Windham G.L., Willcox M.C., Abbas H.K. (2005). Quantitative trait loci contributing resistance to aflatoxin accumulation in the maize inbred Mp313E. Crop Sci..

[153] Brown R.L., Chen Z.-Y., Warburton M., Luo M., Menkir A., Fakhoury A., Bhatnagar D. (2010). Discovery and characterization of proteins associated with aflatoxin-resistance: Evaluating their potential as breeding markers. Toxins.

[154] Chen Z.-Y., Brown R.L., Damann K.E., Cleveland T.E. (2007). Identification of maize kernel endosperm proteins associated with resistance to aflatoxin contamination by *Aspergillus flavus*. Phytopathology.

[155] Jaiswal R., Kuhnert N. (2014). Identification and characterization of the phenolic glycosides of *Lagenaria siceraria* Stand. (Bottle Gourd) fruit by liquid chromatography–tandem mass spectrometry. J. Agric. Food Chem..

[156] Okoth S., Rose L.J., Ouko A., Beukes I., Sila H., Mouton M., Flett B.C., Makumbi D., Viljoen A. (2017). Field evaluation of resistance to aflatoxin accumulation in maize inbred lines in Kenya and South Africa. J. Crop Improv..

[157] Warnatzsch E.A., Reay D.S., Leggieri M.C., Battilani P. (2020). Climate change impact on aflatoxin contamination risk in malawi’s maize crops. Front. Sustain. Food Syst..

[158] Zafar S. (2019). Grain Yield and Nutritional Quality of Commercial Maize (*Zea mays* L.) Genotypes: *Aspergillus* Exposure and Aflatoxin Contamination. Ph.D. Thesis.

[159] Zhang H., Dong M., Yang Q., Apaliya M.T., Li J., Zhang X. (2016). Biodegradation of zearalenone by *Saccharomyces cerevisiae*: Possible involvement of ZEN responsive proteins of the yeast. J. Proteom..

[160] Marshall H., Meneely J.P., Quinn B., Zhao Y., Bourke P., Gilmore B.F., Zhang G., Elliott C.T. (2020). Novel decontamination approaches and their potential application for post-harvest aflatoxin control. Trends Food Sci. Technol..

[161] Matumba L., Van Poucke C., Njumbe Ediage E., Jacobs B., De Saeger S. (2015). Effectiveness of hand sorting, flotation/washing, dehulling and combinations thereof on the decontamination of mycotoxin-contaminated white maize. Food Addit. Contam. Part A.

[162] Pearson T.C., Wicklow D.T., Pasikatan M.C. (2004). Reduction of aflatoxin and fumonisin contamination in yellow corn by high-speed dual-wavelength sorting. Cereal Chem..

[163] Mustapha M.B., Bousselmi M., Jerbi T., Bettaïeb N.B., Fattouch S. (2014). Gamma radiation effects on microbiological, physico-chemical and antioxidant properties of Tunisian millet (*Pennisetum glaucum* LR Br.). Food Chem..

[164] Silva M.V., Janeiro V., Bando E., Machinski M. (2015). Occurrence and estimative of aflatoxin M1 intake in UHT cow milk in Paraná State, Brazil. Food Control..

[165] Wang S.-Q., Huang G.-Q., Li Y.-P., Xiao J.-X., Zhang Y., Jiang W.-L. (2015). Degradation of aflatoxin B 1 by low-temperature radio frequency plasma and degradation product elucidation. Eur. Food Res. Technol..

[166] Markov K., Mihaljević B., Domijan A.-M., Pleadin J., Delaš F., Frece J. (2015). Inactivation of aflatoxigenic fungi and the reduction of aflatoxin B1 in vitro and in situ using gamma irradiation. Food Control.

[167] Ferreira C.D., Lang G.H., da Silva Lindemann I., da Silva Timm N., Hoffmann J.F., Ziegler V., de Oliveira M. (2021). Postharvest UV-C irradiation for fungal control and reduction of mycotoxins in brown, black, and red rice during long-term storage. Food Chem..

[168] Wang B., Mahoney N.E., Pan Z., Khir R., Wu B., Ma H., Zhao L. (2016). Effectiveness of pulsed light treatment for degradation and detoxification of aflatoxin B1 and B2 in rough rice and rice bran. Food Control.

[169] Milani J., Maleki G. (2014). Effects of processing on mycotoxin stability in cereals. J. Sci. Food Agric..

[170] Milani J., Seyed Nazari S.S., Bamyar E., Maleki G. (2018). Effect of bread making process on aflatoxin level changes. J. Chem. Health Risks.

[171] Stoloff L., Trucksess M.W. (1981). Effect of boiling, frying, and baking on recovery of aflatoxin from naturally contaminated corn grits or cornmeal. J. Assoc. Off. Anal. Chem..

[172] Torres P., Guzmán-Ortiz M., Ramírez-Wong B. (2001). Revising the role of pH and thermal treatments in aflatoxin content reduction during the tortilla and deep frying processes. J. Agric. Food Chem..

[173] Suhem K., Matan N., Nisoa M., Matan N. (2013). Inhibition of *Aspergillus flavus* on agar media and brown rice cereal bars using cold atmospheric plasma treatment. Int. J. Food Microbiol..

[174] Shi H., Ileleji K., Stroshine R.L., Keener K., Jensen J.L. (2017). Reduction of aflatoxin in corn by high voltage atmospheric cold plasma. Food Bioprocess Technol..

[175] Rychlik M., Humpf H.-U., Marko D., Dänicke S., Mally A., Berthiller F., Klaffke H., Lorenz N. (2014). Proposal of a comprehensive definition of modified and other forms of mycotoxins including “masked” mycotoxins. Mycotoxin Res..

[176] Suman M., Generotti S. (2015). Transformation of mycotoxins upon food processing: Masking, binding and degradation phenomena. Masked Mycotoxins in Food: Formation, Occurrence Toxicological Relevance.

[177] Lagogianni C., Tsitsigiannis D. (2018). Effective chemical management for prevention of aflatoxins in maize. Phytopathol. Mediterr..

[178] Shi H.U., Stroshine R.L., Ileleji K. (2017). Determination of the relative effectiveness of four food additives in degrading aflatoxin in distillers wet grains and condensed distillers solubles. J. Food Prot..

[179] Yu Y., Shi J., Xie B., He Y., Qin Y., Wang D., Shi H., Ke Y., Sun Q. (2020). Detoxification of aflatoxin B1 in corn by chlorine dioxide gas. Food Chem..

[180] Luo X., Wang R., Wang L., Li Y., Bian Y., Chen Z. (2014). Effect of ozone treatment on aflatoxin B1 and safety evaluation of ozonized corn. Food Control.

[181] Savi G.D., Piacentini K.C., Scussel V.M. (2015). Ozone treatment efficiency in *Aspergillus* and *Penicillium* growth inhibition and mycotoxin degradation of stored wheat grains (*Triticum aestivum* L.). J. Food Process. Preserv..

[182] Aleksić M., Stanisavljević D., Smiljković M., Vasiljević P., Stevanović M., Soković M., Stojković D. (2019). Pyrimethanil: Between efficient fungicide against *Aspergillus* rot on cherry tomato and cytotoxic agent on human cell lines. Ann. Appl. Biol..

[183] Mateo E.M., Gómez J.V., Gimeno-Adelantado J.V., Romera D., Mateo-Castro R., Jiménez M. (2017). Assessment of azole fungicides as a tool to control growth of Aspergillus flavus and aflatoxin B1 and B2 production in maize. Food Addit. Contam. Part A.

[184] Ferrigo D., Mondin M., Scopel C., Dal Maso E., Stefenatti M., Raiola A., Causin R. (2019). Effects of a prothioconazole-and tebuconazole-based fungicide on Aspergillus flavus development under laboratory and field conditions. Eur. J. Plant Pathol..

[185] Masiello M., Somma S., Haidukowski M., Logrieco A.F., Moretti A. (2020). Genetic polymorphisms associated to SDHI fungicides resistance in selected *Aspergillus flavus* strains and relation with aflatoxin production. Int. J. Food Microbiol..

[186] Magnoli K., Benito N., Carranza C., Aluffi M., Magnoli C., Barberis C. (2021). Effects of chlorpyrifos on growth and aflatoxin B1 production by *Aspergillus* section Flavi strains on maize-based medium and maize grains. Mycotoxin Res..

[187] Mannaa M., Oh J.Y., Kim K.D. (2017). Biocontrol activity of volatile-producing *Bacillus megaterium* and *Pseudomonas protegens* against *Aspergillus flavus* and aflatoxin production on stored rice grains. Mycobiology.

[188] Shetty P.H., Hald B., Jespersen L. (2007). Surface binding of aflatoxin B1 by *Saccharomyces cerevisiae* strains with potential decontaminating abilities in indigenous fermented foods. Int. J. Food Microbiol..

[189] Lagogianni C.S., Tsitsigiannis D.I. (2019). Effective biopesticides and biostimulants to reduce aflatoxins in maize fields. Front. Microbiol..

[190] Sivparsad B.J., Laing M.D. (2016). Pre-harvest silk treatment with *Trichoderma harzianum* reduces aflatoxin contamination in sweetcorn. J. Plant Dis. Prot..

[191] Hruska Z., Rajasekaran K., Yao H., Kinkaid R., Darlington D., Brown R.L., Bhatnagar D., Cleveland T.E. (2014). Co-inoculation of aflatoxigenic and non-aflatoxigenic strains of *Aspergillus flavus* to study fungal invasion, colonization, and competition in maize kernels. Front. Microbiol..

[192] Kumar P., Mahato D.K., Gupta A., Pandhi S., Mishra S., Barua S., Tyagi V., Kumar A., Kumar M., Kamle M. (2022). Use of essential oils and phytochemicals against the mycotoxins producing fungi for shelf-life enhancement and food preservation. Int. J. Food Sci. Technol..

[193] Elaigwu M., Oluma H.O.A., Aguoru C.U., Onekutu A. (2020). Screening and phytochemical analysis of some plants extracts against aflatoxin producing fungi in sesame, Benue State, Nigeria. Am. J. Plant Sci..

[194] Kavitha K., Vijaya N., Krishnaveni A., Arthanareeswari M., Rajendran S., Al-Hashem A., Subramania A. (2020). Nanomaterials for antifungal applications. Nanotoxicity.

[195] Tohidi B., Rahimmalek M., Trindade H. (2019). Review on essential oil, extracts composition, molecular and phytochemical properties of *Thymus* species in Iran. Ind. Crops Prod..

[196] Makhuvele R., Naidu K., Gbashi S., Thipe V.C., Adebo O.A., Njobeh P.B. (2020). The use of plant extracts and their phytochemicals for control of toxigenic fungi and mycotoxins. Heliyon.

[197] López-Meneses A.K., Plascencia-Jatomea M., Lizardi-Mendoza J., Fernández-Quiroz D., Rodríguez-Félix F., Mouriño-Pérez R.R., Cortez-Rocha M.O. (2018). *Schinus molle* L. essential oil-loaded chitosan nanoparticles: Preparation, characterization, antifungal and anti-aflatoxigenic properties. LWT.

[198] Da Silva Bomfim N., Kohiyama C.Y., Nakasugi L.P., Nerilo S.B., Mossini S.A.G., Romoli J.C.Z., Graton Mikcha J.M., Abreu Filho B.A.d., Machinski M. (2020). Antifungal and antiaflatoxigenic activity of rosemary essential oil (*Rosmarinus officinalis* L.) against *Aspergillus flavus*. Food Addit. Contam. Part A.

[199] Kujur A., Kumar A., Yadav A., Prakash B. (2020). Antifungal and aflatoxin B1 inhibitory efficacy of nanoencapsulated *Pelargonium graveolens* L. essential oil and its mode of action. LWT.

[200] Kocić-Tanackov S., Dimić G., Jakšić S., Mojović L., Djukić-Vuković A., Mladenović D., Pejin J. (2019). Effects of caraway and juniper essential oils on aflatoxigenic fungi growth and aflatoxins secretion in polenta. J. Food Process. Preserv..

[201] García-Díaz M., Patiño B., Vázquez C., Gil-Serna J. (2019). A novel niosome-encapsulated essential oil formulation to prevent *Aspergillus flavus* growth and aflatoxin contamination of maize grains during storage. Toxins.

[202] Nasseri M., Golmohammadzadeh S., Arouiee H., Jaafari M.R., Neamati H. (2016). Antifungal activity of *Zataria multiflora* essential oil-loaded solid lipid nanoparticles in-vitro condition. Iran. J. Basic Med. Sci..

[203] García-Díaz M., Gil-Serna J., Patiño B., García-Cela E., Magan N., Medina Á. (2020). Assessment of the effect of *Satureja montana* and *Origanum virens* essential oils on *Aspergillus flavus* growth and aflatoxin production at different water activities. Toxins.

[204] Hossain F., Follett P., Salmieri S., Vu K.D., Fraschini C., Lacroix M. (2019). Antifungal activities of combined treatments of irradiation and essential oils (EOs) encapsulated chitosan nanocomposite films in *in vitro* and *in situ* conditions. Int. J. Food Microbiol..

[205] Das S., Singh V.K., Dwivedy A.K., Chaudhari A.K., Upadhyay N., Singh A., Dubey N.K. (2020). Fabrication, characterization and practical efficacy of *Myristica fragrans* essential oil nanoemulsion delivery system against postharvest biodeterioration. Ecotoxicol. Environ. Saf..

[206] Do K.H., An T.J., Oh S.-K., Moon Y. (2015). Nation-based occurrence and endogenous biological reduction of mycotoxins in medicinal herbs and spices. Toxins.

[207] Chen C., Long L., Zhang F., Chen Q., Chen C., Yu X., Liu Q., Bao J., Long Z. (2018). Antifungal activity, main active components and mechanism of *Curcuma longa* extract against *Fusarium graminearum*. PLoS ONE.

[208] Hu Y., Luo J., Kong W., Zhang J., Logrieco A.F., Wang X., Yang M. (2015). Uncovering the antifungal components from turmeric (*Curcuma longa* L.) essential oil as *Aspergillus flavus* fumigants by partial least squares. RSC Adv..

[209] Oliveira G.d.S., Nascimento S.T., Dos Santos V.M., Silva M.G. (2020). Clove essential oil in the sanitation of fertile eggs. Poult. Sci..

[210] Kocić-Tanackov S.D., Dimić G.R. (2013). Antifungal activity of essential oils in the control of food-borne fungi growth and mycotoxin biosynthesis in food. Metabolism.

[211] Mutlu-Ingok A., Devecioglu D., Dikmetas D.N., Karbancioglu-Guler F., Capanoglu E. (2020). Antibacterial, antifungal, antimycotoxigenic, and antioxidant activities of essential oils: An updated review. Molecules.

[212] Dwivedy A.K., Kumar M., Upadhyay N., Prakash B., Dubey N.K. (2016). Plant essential oils against food borne fungi and mycotoxins. Curr. Opin. Food Sci..

[213] Bluma R., Amaiden M.R., Daghero J., Etcheverry M. (2008). Control of *Aspergillus* section Flavi growth and aflatoxin accumulation by plant essential oils. J. Appl. Microbiol..

[214] Restuccia C., Conti G.O., Zuccarello P., Parafati L., Cristaldi A., Ferrante M. (2019). Efficacy of different citrus essential oils to inhibit the growth and B1 aflatoxin biosynthesis of *Aspergillus flavus*. Environ. Sci. Pollut. Res..

[215] Jantapan K., Poapolathep A., Imsilp K., Poapolathep S., Tanhan P., Kumagai S., Jermnak U. (2017). Inhibitory effects of Thai essential oils on potentially aflatoxigenic *Aspergillus parasiticus* and *Aspergillus flavus*. Biocontrol Sci..

[216] Songsamoe S., Matan N., Matan N. (2017). Antifungal activity of *Michelia alba* oil in the vapor phase and the synergistic effect of major essential oil components against *Aspergillus flavus* on brown rice. Food Control.

[217] Basak S., Guha P. (2018). A review on antifungal activity and mode of action of essential oils and their delivery as nano-sized oil droplets in food system. J. Food Sci. Technol..

[218] Zhang C., Zhao J., Famous E., Pan S., Peng X., Tian J. (2021). Antioxidant, hepatoprotective and antifungal activities of black pepper (*Piper nigrum* L.) essential oil. Food Chem..

[219] Das S., Singh V.K., Dwivedy A.K., Chaudhari A.K., Dubey N.K. (2021). Nanostructured *Pimpinella anisum* essential oil as novel green food preservative against fungal infestation, aflatoxin B1 contamination and deterioration of nutritional qualities. Food Chem..

[220] Upadhyay N., Singh V.K., Dwivedy A.K., Chaudhari A.K., Dubey N.K. (2021). Assessment of nanoencapsulated *Cananga odorata* essential oil in chitosan nanopolymer as a green approach to boost the antifungal, antioxidant and in situ efficacy. Int. J. Biol. Macromol..

[221] Chaudhari A.K., Singh V.K., Das S., Prasad J., Dwivedy A.K., Dubey N.K. (2020). Improvement of in vitro and in situ antifungal, AFB1 inhibitory and antioxidant activity of *Origanum majorana* L. essential oil through nanoemulsion and recommending as novel food preservative. Food Chem. Toxicol..

[222] Nerilo S.B., Romoli J.C.Z., Nakasugi L.P., Zampieri N.S., Mossini S.A.G., Rocha G.H.O., Gloria E.M.d., Abreu B.A.d., Machinski M. (2020). Antifungal activity and inhibition of aflatoxins production by *Zingiber officinale* Roscoe essential oil against *Aspergillus flavus* in stored maize grains. Cienc. Rural.

